# Ecotoxicity of Nitrated Monoaromatic Hydrocarbons in Aquatic Systems: Emerging Risks from Atmospheric Deposition of Biomass Burning and Anthropogenic Aerosols

**DOI:** 10.3390/toxics13121037

**Published:** 2025-11-30

**Authors:** Saranda Bakija Alempijević, Slađana Strmečki, Ivan Mihaljević, Sanja Frka, Jelena Dragojević, Ivana Jakovljević, Tvrtko Smital

**Affiliations:** 1 Division for Marine and Environmental Research, Ruđer Bošković Institute, Bijenička 54, 10 000 Zagreb, Croatia; saranda.bakija.alempijevic@irb.hr (S.B.A.); frka@irb.hr (S.F.); jelena.dragojevic@irb.hr (J.D.); smital@irb.hr (T.S.); 2 Institute for Medical Research and Occupational Health, Ksaverska Cesta 2, 10 000 Zagreb, Croatia; ijakovljevic@imi.hr

**Keywords:** air pollution, aquatic systems, atmospheric aerosols, bioassays, ecotoxicological effects, nitrocatechols, zebrafish (*Danio rerio*)

## Abstract

Nitrated monoaromatic hydrocarbons (NMAHs) are emerging air pollutants commonly found in biomass burning (BB) and anthropogenic aerosols (AA). Despite their frequent deposition into aquatic systems, their ecotoxicity is still poorly understood. This study evaluates the toxicity of BB and AA aerosol extracts and their main NMAH constituents (nitrocatechols, nitrophenols, and nitrosalicylic acids) using *in vitro* (cellular uptake, cytotoxicity) and *in vivo* (algal growth inhibition, zebrafish embryo development) bioassays. Polar aerosol extracts showed higher toxicity than nonpolar ones, with stronger interaction via zebrafish organic anion Oatp1d1 than organic cation Oct1 transporter, indicating selective uptake. NMAHs and their relevant mixtures showed similar toxicity patterns as BB water extract, so NMAHs were identified as contributors to aerosol toxicity. Nitrocatechols stand out for their toxicity, showing the highest chronic toxicity in algae (IC_50_: 0.6–1.1 mg/L) and acute cytotoxicity in fish cells (IC_50_: 2.0–4.1 mg/L), possibly because they dominated the NMAHs composition of aerosols (BB: 80.6%; AA: 79.8%). Sublethal NMAH concentrations caused developmental disorders and altered lipid homeostasis in zebrafish embryos, indicating early physiological stress on higher organisms. These findings reveal NMAHs as significant ecotoxic components of BB and AA emissions which may pose an increasing threat to aquatic ecosystems following atmospheric deposition.

## 1. Introduction

Air pollution poses serious environmental risks, with atmospheric aerosols among the most concerning pollutants. The chemical composition of aerosols, comprising inorganic ions, organic substances, elemental carbon, and trace metals [1], along with their source and size, critically determines their ecotoxicity [2]. For larger areas such as the atmosphere, there are extensive studies that highlight modern methods of determination of different pollutants and their precursors [3]. Since organic matter can comprise up to 90% of fine aerosols (particle aerodynamic diameter, *d* < 2.5 µm, PM_2.5_), it significantly influences aerosol ecotoxicity, especially when originating from biomass burning (BB), fossil fuel combustion, and industrial processes [4,5]. Biomass burning, whether in the form of open vegetation fire or indoor biofuel combustion, is not just a source of pollutants, it initiates complex chemical and physical processes involving atmospheric chemistry, pollutant transport and transformation, climate forcing, and ecological disruption [6]. These processes extend far beyond the fire zone, linking local combustion events to global environmental and health challenges. Aquatic systems represent an important sink for deposited atmospheric pollutants.

Recently, nitrated monoaromatic hydrocarbons (NMAHs) such as nitrophenols (NPs) and nitrocatechols (NCs) have gained attention as emerging atmospheric pollutants. They have been detected in aerosols across the Europe [7,8,9,10,11], South America [8] and Asia [12,13] at concentrations ranging from 0.3 to 520.0 ng/m^3^ of air. Trace levels of 4-nitrophenol (4NP; 8–13 ng/L) have been detected in freshwater and snow in Antarctica [14], pointing to their global distribution. Their quantity in aerosols varies seasonally, with higher concentrations in autumn and winter due to enhanced wood heating [9] and industrial activities in urban regions [8]. At the global level, BB represents one of the most important sources of atmospheric NMAHs, and 4-nitrocatechol (4NC) and methyl-nitrocatechol isomers have been identified as major tracers for BB and aged anthropogenic aerosols [7].

It is known that 74% of the total concentration of NMAHs detected in PM_15.6_ (*d* < 15.6 µm) belongs to the submicron aerosol size fraction [9], indicating their high potential for long-range transport and long atmospheric residence. NMAHs are found in primary emitted organic particles and are also generated through secondary atmospheric processes during aerosol aging [11,15]. Secondary chemical reactions include nitration and (photo) oxidation of volatile organic compounds [7] and reactions triggered by radicals [16].

Furthermore, NMAHs can make up to 3% of aerosol water-soluble organic carbon (WSOC) [8,9] and are efficiently scavenged by wet deposition of aerosols into various aquatic systems with daily rates up to 76 µg NMAHs/L [10,17]. In addition to atmospheric input, NPs enter waters through direct terrestrial input [18], or they can be formed in situ in seawater as secondary pollutants through phenol transformation [19].

The ecotoxicological studies on the effects of NMAHs on aquatic organisms have primarily focused on NPs, using different target organisms and bioassays. These studies have identified the structure [20], concentration [21], and environmental conditions [22] as key factors influencing NPs toxicity to marine and freshwater organisms. Moreover, various morphological deformities have been observed in zebrafish embryos exposed to sublethal concentrations of NPs [21] and NCs [23]. The observed toxicities of NPs are attributed to their action as uncoupling agents in oxidative phosphorylation and alteration of cellular metabolism [17,21].

Consequently, the U.S. Environmental Protection Agency (EPA) recognized the hazardous nature of some NPs, categorizing 2,4-dinitrophenol (2,4DNP), 2-nitrophenol (2NP), and 4-nitrophenol (4NP) as toxic and priority pollutants under the Clean Water Act [24] and 2,4DNP and 4NP as hazardous air pollutants under the Clean Air Act [25]. However, neither NCs nor other NPs have been officially classified as harmful substances. The absence of such classification highlights the lack of systematic studies which correlate concentrations of NMAHs in aerosols with their (eco)toxicity.

To address this gap, we investigated the acute and chronic toxicity of aerosols emitted from two major sources of NMAHs: simulated biomass burning (sBB) and ambient anthropogenic (AA) aerosols. Acute toxicity assessments provide immediate insights into the potential hazards of NMAHs, while chronic toxicity evaluations are essential for understanding prolonged environmental and biological impacts. In this study, the initial toxicokinetic step, i.e., the uptake of xenobiotics (phase 0 of absorption, distribution, metabolism, and excretion (ADME) processes) was evaluated by investigating the interaction with zebrafish membrane uptake transporters Oatp1d1 and Oct1. These transporters are critical for mediating the entry mechanisms of organic anions and cations into zebrafish cells, influencing their bioavailability and subsequent toxicity. Subsequently, phase I metabolism, which involves enzymatic modification to increase compound solubility and facilitate elimination, was assessed using the EROD assay, which specifically measures cytochrome P450 enzyme activity, a primary indicator of the organism’s metabolic response to xenobiotic exposure. We also applied the mentioned bioassays to investigate toxicities of individual NMAHs and their environmentally relevant water mixtures, thereby providing a basis for understanding their harmful potential under realistic aerosol exposure scenarios. Additionally, the Fish Embryo Acute Toxicity test was conducted to provide direct evidence of NMAHs developmental effects such as mortality, morphological malformations, and behavioral disturbances in zebrafish embryos. Finally, alterations in the lipid profiles of zebrafish embryos exposed to NMAHs were examined, given that lipid composition serves as a reliable biomarker of early cellular stress and membrane integrity disruption. Together, these integrated tests allowed us to link sBB and AA aerosol ecotoxicity directly to the chemical composition of NMAHs.

## 2. Materials and Methods

### 2.1. Chemicals

Unless otherwise stated, all chemicals used were purchased from Sigma-Aldrich, St Louis, MO, USA. Milli-Q water (18.2 MW cm) was prepared in a filtration apparatus (Millipore, Billerica, MA, USA).

### 2.2. BB and AA Aerosols Collection and Leaching

Particles in the PM_10_ size range (≤10 µm), sufficiently small to remain airborne and undergo long-range transport, yet large enough to carry significant amounts of organic pollutants, such as NMAHs associated with BB and anthropogenic emissions [9], have been chosen for the analysis.

The PM_10_ aerosols from the simulated burning of dry oak firewood (sBB aerosols) were collected on four pre-baked (4 h at 450 °C) quartz fibre filters (154 cm^2^; Whatman, Little Chalfont, UK) in a high-volume sampler (Kalman System, Budapest, Hungary). The firewood logs were burned in a Servant S114 cast-iron stove with a heating capacity of 5 kW. The stove was connected to an approximately 8 m high chimney with an internal diameter of 12 cm. At the chimney outlet, a small portion of the flue gases was directed into a dilution unit that housed the sampling inlet of the high-volume aerosol sampler. There was no control of oxygen supply and the combustion temperature was not directly measured; however, typical firewood combustion in cast-iron stoves reaches approximately 600–900 °C in the combustion chamber, while flue gas temperatures at the chimney outlet commonly range between 150–300 °C. Aerosol samples were collected during flaming, weakly flaming, and smoldering phases, as the different temperatures of each phase yielded distinct types and fractions of NMAHs [26]. The filter samples were placed in pre-baked Al foils and stored at −20 °C. The four sections (17.66 mg of particles), each from one sBB filter, were cut into pieces and jointly extracted three times using 15 mL of H_2_O each time. Then, three new sets of four sBB sections were prepared for similar separate extractions using methanol (MeOH), hexane (Hex), and dichloromethane (DCM). The extractions were carried out by ultrasonic agitation for 15 min, followed by storage at 4 °C for 24 h. The solutions obtained were filtered through 0.7 µm filters (GF/F, Whatman) to remove suspended insoluble particles and filter debris. The water extracts were then freeze-dried (5.5 Pa, −48 °C) using benchtop freeze dryer (Labconco, Kansas City, MO, USA), while the organic solvents were evaporated using a Rotavapor^®^ R-100 (Buchi Labortechnik, Flawil, Switzerland) followed by a gentle nitrogen stream.

The PM_10_ AA aerosol samples (14 filters) exposed to various anthropogenic pollution sources (fossil fuel combustion, industrial activities) were collected in the continental part of Croatia on pre-combusted (450 °C for 4 h) quartz fibre filters (*d* = 47 mm; Pall Life Sciences, Port Washington, NY, USA) using an automatic sequential low-volume sampler PNS 18T-DM-3.1 (Comde-Derenda, Stahnsdorf, Germany) at a volume airflow rate of 2.3 m^3^/h. The filter sections (66.24 mg of particles) were combined, chopped, and leached in 0.8 L of MQ water by ultrasound and stored at 4 °C for 24 h. The solution was then filtered (GF/F, Whatman, pore size of 0.7 µm), while 30 mL was separated (2.5 mg of particles) and lyophilized.

Dry aerosol extracts of sBB (H_2_O, MeOH, Hex, DCM) and AA (H_2_O) were used for LC-MS/MS quantification of 12 NMAHs, while other similar sets of extracts were prepared for bioassays.

### 2.3. Quantification of NMAHs in sBB and AA Aerosol Extracts

Commercially available 4NC, 4-methyl-5-nitrocatechol (4M5NC, Santa Cruz Biotechnology, Dallas, TX, USA), 3-methyl-5-nitrocatechol (3M5NC, Atomax Chemicals, Shenzhen, China), 4NP, 2-methyl-4-nitrophenol (2M4NP), 3-methyl-4-nitrophenol (3M4NP), 2,4DNP, 4-nitroguaiacol (4NG), 4-nitrosyringol (4NS), 3-nitrosalycilic acid (3NSA) and 5-nitrosalycilic acid (5NSA), as well as synthesized 3-methyl-4-nitrocatechol (3M4NC, purified and identified in [27]) were used as standards.

For LC-MS/MS quantification of 12 NMAHs in aerosol extracts, filter sections were spiked with 100 ng of picric acid (internal standard), chopped, and extracted as described in Section 2.2. Different dry aerosol extracts, as well as corresponding NMAHs standards, were (re)dissolved in 500 µL of injection solvent and subsequently filtered through 0.22 µm syringe filters (Whatman; GE Healthcare, Little Chalfont, UK). The injection solvent consisted of 7.5 mM ammonium formate buffer, pH 3 (made of ammonium formate and formic acid, LC-MS eluent additives, Fluka, Seelze, Germany) and 285 µM EDTA (99.995%).

The analysis was performed using a triple quadrupole LC/MS-MS (Agilent Technologies, Santa Clara, CA, USA) in conjunction with an UltiMate 3000 UHPLC system (Thermo Scientific, Waltham, MA, USA). The Atlantis T3 (3.0 × 150 mm^2^, 3 µm particle size; Waters, Milford, MA, USA) HPLC column and the Atlantis T3 guard column (3.0 × 10 mm^2^, 3 µm particle size; Waters) were used with a flow rate of 0.3 mL/min. A mixture of tetrahydrofuran (Chromasolv Plus, HPLC grade, 99.9%, Fluka), MeOH (Chromasolv LC-MS grade, 99.9%, Fluka, Germany), and water (15/30/55, *v*/*v*/*v*) with 7.5 mM ammonium formate buffer, pH 3, was used as the mobile phase. The injection volume was 10 µL and column temperature was set to 30 °C. The gradient elution program is described in [27]. The determination of NMAHs in the PM filter samples was done using a validated analytical procedure described in [9,11,28,29]. The performance of the applied method was evaluated with respect to linearity, and detection (LOD) and quantification (LOQ) limits of target analytes [10]. All calibration plots were constructed with six calibration points (R^2^ = 0.9948–1.0). Three filter blanks were prepared during sample extraction and treated in the same way as the sBB and AA samples. These blanks were retrieved and processed together with all other samples. For data correction, the mean of blank values was subtracted from the corresponding sample values.

### 2.4. Bioassays

Dry aerosol extracts and powdered model NMAHs were (re)dissolved in dimethyl sulfoxide for use in bioassays. Aerosol extract concentrations were calculated based on the particle mass collected on the respective filter sections (details in Supplementary Table S1). For individual NMAHs, serial dilutions spanning from no-effect levels to concentrations eliciting maximum response were prepared to generate concentration–response curves. Two model mixtures were tested: Σ_10_NMAHs (25.8 mg/L), formulated to match the mass of ten NMAHs detected in sBB_H2O_, and Σ_5_NMAHs (24.9 mg/L), composed of the five most abundant compounds representing 96.5% of the total NMAHs mass. All exposures were conducted in two to three independent experiments, each with duplicate or triplicate determinations. Measurements were recorded using a microplate reader (Infinite M200, Tecan, Salzburg, Austria).

#### 2.4.1. Interaction with Phase 0 Membrane Transporters Oct1 and Oatp1d1

For the interaction of aerosol extracts and NMAHs with phase 0 membrane transporters Oct1 and Oatp1d1 we used stable transfected cell lines Flp-In-293-drOct1 [30] and Flp-In-293-drOatp1d1 [31], respectively. The principle of inhibition assays was based on the co-exposure of transfected cells and the mock cells (transfected with an empty vector; blank control). The details on the exposure protocols are given in Supplementary Materials. For the assays, 4-(4-(dimethylamino)styryl)-N-methylpyridinium iodide (ASP+, 0.5 or 1 µM) was used as the fluorescent substrate for Oct1, and Lucifer yellow (LY, 50 µM) for Oatp1d1. The LY fluorescence was measured at 425/540 nm, which corresponds to its optimal excitation at ~425 nm and emission near ~530–540 nm. ASP+ fluorescence was recorded at 450/590 nm for aerosol extract experiments (1 µM ASP+; Supplementary Table S1) and at 470/605 nm for model NMAHs (0.5 µM ASP+; Supplementary Table S3); both pairs fall within the known excitation (450–470 nm) and emission (590–615 nm) maxima of ASP+ and were selected based on prior optimization to maximize signal-to-noise ratio in the respective matrices. The fluorescence of mock cells was subtracted from that of transfected cells and the reaction mixture without analytes served as a negative control.

#### 2.4.2. Interaction with Phase I Cellular Detoxification: The EROD Test

The EROD bioassay measures the activity of ethoxy resorufin-O-deethylase, a key enzyme in the cytochrome P-4501A1-mediated monooxygenase system (CYP1A1), which is part of phase I of the cellular detoxification. The principle of the assay is the conversion of ethoxy-resorufin, a non-fluorescent substrate of CYP1A1, into a fluorescent resorufin by the removal of its ethoxy group when it enters the cell. The EROD assay was conducted in PLHC-1 cells using the standardized protocol described in [32] which specifies physiological pH conditions. After incubating the cells with aerosol extracts or model NMAHs according to the procedure described in Supplementary Material, the fluorescence of resorufin was measured at 535/590 nm at 1-min intervals for 10 min following substrate addition. The 2,3,7,8-tetrachlorodibenzo-p-dioxin was used as a model CYP1A1 inducer. The measured plates were then frozen at −20 °C and the protein content was subsequently quantified using the fluorescamine (Alfa Aesar, Ward Hill, MA, USA) assay with bovine serum albumin as the standard [33]. Enzyme activity was expressed in pmol resorufin/min mg of protein.

#### 2.4.3. Acute Cytotoxicity: The MTT Assay

The acute cytotoxicity was evaluated on PLHC-1 fish cells using the MTT (3-(4,5-dimethyl-2-thiazolyl)-2,5-diphenyl-2H-tetrazolium bromide, 98%; Alfa Aesar, Ward Hill, MA, USA) colorimetric reduction assay [34]. Briefly, a yellow tetrazole is reduced to purple formazan salts in living cells, and the salts are then dissolved in isopropanol. The absorbance of the resulting solution was measured at 578 nm with a reference filter at 750 nm. Additional details are provided in Supplementary Materials. The cytotoxicity was determined by comparing mitochondrial activity to untreated control cells, with cyclosporine A as a positive control.

#### 2.4.4. Chronic Toxicity: The AlgaeTox Assay

The chronic toxicity was tested on the unicellular freshwater green alga *Scenedesmus subspicatus* using the standardized method ISO 8692:2004. The algae stock culture was maintained in a growth medium with macronutrients (NH_4_, Mg, Ca, Mg, PO_4_), trace elements (B, Mn, Zn, Co, Cu, Mo), Fe-EDTA and NaHCO_3_ at 24 ± 2 °C under continuous light (60–120 µmol/m^2^ s). The inoculum (1 × 10^4^ cells/mL) of exponentially growing algal cells was exposed to our analytes for 96 h in 96-well microplate. Algae density was determined by measuring the chlorophyll *a* fluorescence at 440/680 nm at the start and end of the incubation and results were expressed as percentage cell viability vs. test concentration and compared to a blank sample. A positive control with K_2_Cr_2_O_7_ (Alfa Aesar, Ward Hill, MA, USA) and the blank sample were grown under identical conditions.

#### 2.4.5. Zebrafish Maintenance and Embryotoxicity Assay

The ABO wild-type zebrafish line (European Zebrafish Resource Centre, Karlsruhe, Germany) was raised under standard conditions, with a 14 h light and 10 h dark cycle and a constant water temperature of 27–28 °C. Fish were fed an appropriately sized standard diet (Gemma Micro, Skretting, Stavanger, Norway). To obtain embryos, 1–1.5-year-old zebrafish were spawned in the morning and fertilized embryos transferred to a Petri dish containing the E3 medium (5 mM NaCl, 0.17 mM KCl, 0.33 mM CaCl_2_, 0.33 mM MgSO_4_) and then incubated at 28 °C under the same light-dark cycle as the adult fish. The development of the embryos was monitored using a stereomicroscope (Motic AE31E, Motic, Barcelona, Spain) and stages were determined based on established methods [35].

Fish Embryo Acute Toxicity test on zebrafish (*Danio rerio*) embryos was performed according to the modified guidelines [36]. Freshly fertilized eggs were separated from unfertilized ones, placed in a standard 24-well plate, and incubated with a range of concentrations (4.6–203.0 mg/L) of model NMAHs, diluted in 0.5 mL E3 medium. The exposure occurred in the dark in an incubator at 28 °C. Each concentration was tested in duplicate, with 20 embryos (10 per well) per concentration. The embryos exposed to the highest used concentration of dimethyl sulfoxide (<0.1%) served as negative controls, with a survival rate above 90%. Sublethal (morphological) abnormalities were followed at 24, 48 and 72 hpf using a stereomicroscope (Motic AE31E, Motic, Barcelona, Spain).

### 2.5. Extraction and Detection of Lipids in Zebrafish Embryos Exposed to NMAHs

Zebrafish embryos were exposed in 24-well microplates (13–15 embryos per well, 26–30 total, final volume 2 mL per well) to sublethal concentrations of 2,4DNP (9.2 mg/L), 4NS (5.0 mg/L), 4NG (42.3 mg/L), 3M5NC (42.3 mg/L) or 4NC (38.8 mg/L) during 48 hpf. Two sets of embryos without added NMAHs were used as controls. After exposure, embryos were frozen at −80 °C, freeze-dried, and weighed. Palmitic acid methyl ester (16 µg, internal standard) was added to the embryos and total lipids were extracted using a modified procedure of [37,38]. The embryos were homogenized in 550 µL of H_2_O:MeOH (1:1). Chloroform:MeOH (1:2, 750 µL) and chloroform (250 µL) were then sequentially added, with vortexing for 30 s after each addition. The samples were subsequently incubated at 25 °C for 10 min and centrifuged at 3000× *g* for 5 min. The organic phase containing lipids was separated and immediately evaporated under nitrogen flow. The extracted lipids were stored at −20 °C and redissolved in 1 mL of DCM before analysis.

Lipid classes were separated through five elution steps in the solvents of increasing polarity and analysed using thin layer chromatography–flame ionization detection at Iatroscan MK-VI (Iatron, Tokyo, Japan; air flow 2000 mL/min, hydrogen flow 160 mL/min) [39]. Identified lipid classes relevant for zebrafish embryos [40,41,42] were: free fatty acids (FFA), triacylglycerols (TG), alcohols (ALC), cholesterol (COH), 1,2-diacylglicerols (1,2DG), 1,3-diacylglicerols (1,3DG), wax esters (WE), glycolipids (GL, including monogalactosyldiacylglycerols (MGDG), digalactosyldiacylglycerols (DGDG) and sulfoquinovosyldiacylglycerols (SQDG)), and phospholipids (PL, including phosphatidylglycerol (PG), phosphatidylethanolamine (PE) and phosphatidylcholine (PC)). Lipid classes were quantified using an external standard lipid mixture and expressed as a percentage of embryo dry weight. Each sample was measured twice with a relative standard deviation < 10%. The concentration of total lipids was calculated as the sum of all analysed lipid classes. The results represent mean of two exposure sets.

### 2.6. Data Analysis

Data were processed using Microsoft Office Professional Plus 2019 (Microsoft, Redmond, WA, USA), GraphPad Prism 8 (GraphPad Software, San Diego, CA, USA), or Origin 2018, (OriginLab, Northampton, MA, USA). Bioassays were performed in duplicates or triplicates and concentration-response curves with 95% confidence intervals were generated. The half-maximal inhibitory concentration (IC_50_) was calculated using the sigmoidal four-parameters dose-response model [30]. Statistical analysis was conducted using Student’s *t*-test and one-way ANOVA, while correlations were explored using Pearson correlation analysis, all with significance set at *p* < 0.05.

## 3. Results

### 3.1. Aerosol Extracts

#### 3.1.1. Effects of Aerosol Extracts on Phase 0 and I of Cellular Detoxification

**Figure 1:**
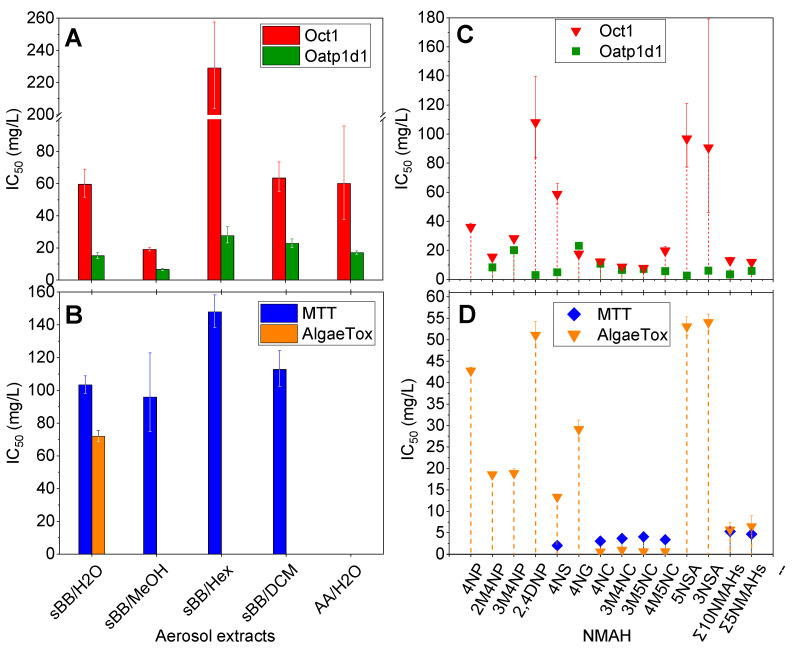
Bioassays performed with: (**A**,**B**) simulated biomass burning (sBB) and ambient anthropogenic (AA) aerosol extracts, and (**C**,**D**) individual NMAHs and their mixtures (Σ_10_NMAHs and Σ_5_NMAHs). (**A**,**C**) Inhibition of zebrafish (*Danio rerio*) organic cation transporter 1 (Oct1) and organic anion-transporting polypeptide 1d1 (Oatp1d1), expressed as half-maximal inhibitory concentrations (IC_50_) for uptake of the fluorescent model substrates ASP+ and Lucifer yellow, respectively. (**B**,**D**) Acute cytotoxicity in PLHC-1 fish cells assessed by MTT assay, and chronic toxicity in the freshwater green alga *Scenedesmus subspicatus* using the AlgaeTox assay, expressed as IC_50_. Vertical bars represent 95% confidence interval for IC_50_.

The IC_50_ values for inhibition of organic cation transporter Oct1 ranged from 19.2 (sBB_MeOH_) to 225.3 mg/L (sBB_Hex_) and were consistently higher than those for organic anion-transporting polypeptide Oatp1d1 (6.6 (sBB_MeOH_)–27.7 mg/L (sBB_Hex_), Figure 1A; Supplementary Figures S1 and S2). Reported higher IC_50_ values for Oct1 inhibition by sBB samples likely reflect the chemical composition of the aerosol extracts. Biomass burning aerosols contain predominantly acidic and oxygenated organic compounds such as phenols, organic acids, nitro-aromatics that are predominantly anionic at physiological pH. They are more compatible with the substrate preferences of Oatp1d1 and therefore inhibit this transporter more efficiently. In contrast, organic cations, which would more effectively compete for Oct1, are present at much lower levels, resulting in weaker Oct1 inhibition. Lower IC_50_ obtained for polar extracts indicated that they extracted higher concentration of compounds that interacted with membrane transporters. Both sBB and AA extracts exhibited stronger interaction of anion transport via Oatp1d1 compared to cation transport via Oct1. Notably, the aqueous AA extract (AA_H2O_) showed a sevenfold stronger interaction with Oatp1d1 than Oct1. These results suggest that under the experimental conditions, negatively charged organic compounds in extracts interacted more potently with membrane transporters than positively charged ones.

In the EROD bioassay, only AA_H2O_ induced CYP1A1, a phase I detoxification enzyme (Supplementary Figure S3), with EC_50_ (half-maximal effective concentration) of 31.8 mg/L, possibly due to a different chemical composition of source specific sBB and AA aerosols, with AA probably containing more specific hydrophobic/lipophilic compounds that induced CYP1A1, as discussed in Section 4.3.

#### 3.1.2. Acute and Chronic Effects of Aerosol Extracts

In the MTT assay, the sBB extracts reduced PLHC-1 cell viability, indicating acute cytotoxicity effects. The IC_50_ values were relatively high across all sBB extracts, with lowest viability observed for sBB_MeOH_ (IC_50_ = 95.9 mg/L) and the highest for sBB_Hex_ (IC_50_ = 148.0 mg/L) (Figure 1B; Supplementary Figure S4), suggesting efficient extraction of cytotoxic organic constituents by more polar solvents. In contrast, the AA_H2O_ did not affect PLHC-1 cells viability within the tested concentration range.

In chronic toxicity assays with *S. subspicatus*, only the sBB_H2O_ inhibited algal growth, with IC_50_ of 71.9 mg/L (Figure 1B; Supplementary Figure S5). In contrast, sBB_MeOH_, sBB_Hex_ and AA_H2O_ stimulated algal growth across the tested concentrations (Supplementary Figure S5). The sBB_DCM_ promoted growth at lower concentrations (up to 220.7 mg/L of particles), but caused inhibition at higher level, with complete loss of viable cell observed at 1766.0 mg/L. Such effects could be due to varying quantity of extracted material, as discussed later.

### 3.2. Individual NMAHs

#### 3.2.1. Concentration of NMAHs in Aerosols Extracts

The pronounced interaction of the water aerosol extracts with phase 0 uptake transporters, along with its clear acute and chronic toxic effect, suggests that polar, hydrophilic organic compounds may be the primary contributors to the observed aerosol toxicity. As NMAHs are water-soluble polar molecules, 12 common atmospherically related NMAHs (Table 1) were quantified in each of the extracts.

**Figure 2:**
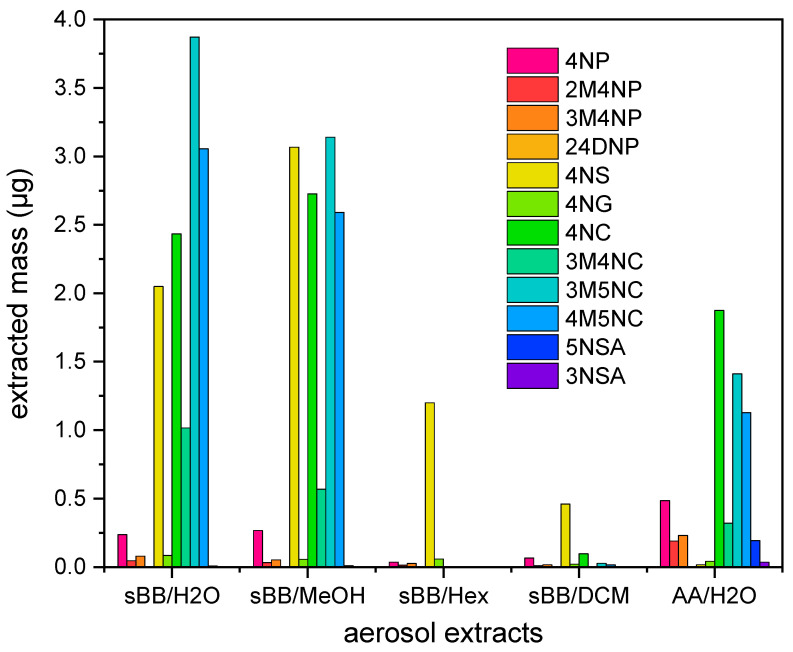
Concentrations of 12 NMAHs in water (H_2_O), methanol (MeOH), hexan (Hex), and dichloromethane (DCM) extracts of simulated biomass burning (sBB) and H_2_O extract of ambient anthropogenic (AA) aerosol determined according to the validated analytical procedure using LC/MS-MS.

The total quantified mass of ten NMAHs in sBB extracts decreased in the order: water (12.9 µg) ≈ methanol (12.5 µg) > hexane (1.3 µg) > dichloromethane (0.7 µg), highlighting NMAHs strong solubility in polar solvents. Neither nitrocatechols (NCs; 4NC, 3M4NC, 3M5NC, 4M5NC) nor nitrosalicylic acid (NSAs) were quantified in the hexane extract. Five compounds (3M5NC, 4M5NC, 4NC, 4NS, and 3M4NC) accounted for 96.5% of total NMAH mass in the sBB_H2O_. Based on these results, AA aerosols were extracted using water only. In AA_H2O_, NCs were also the most abundant compounds (79.8%), followed by NPs (16.3%) and NSAs (3.9%) (Figure 2). The concentration of 2,4DNP was below the quantification limit in all extracts, while 3NSA was detected only in AA_H2O_. This is consistent with previous findings indicating that 2,4DNP and 3NSA typically occur at low concentrations in European ambient aerosols [9,48].

#### 3.2.2. Effects of NMAHs on Phase 0 and I of Cellular Detoxification

All tested NMAHs interfered with ASP+ uptake via Oct1, while for Oatp1d1, all compounds except 4NP showed interaction with LY uptake at concentrations up to 100 mg/L (Supplementary Tables S3 and S4). The IC_50_ values are presented in Figure 1C, with detailed interaction profiles shown in Supplementary Figures S6 (ASP+ substrate, Oct1) and S7 (LY substrate, Oatp1d1). Nitrocatechols, particularly 3M5NC, 3M4NC, and 4NC, exhibited the strongest interactions with Oct1. In contrast, the weakest Oct1 interactions were observed for 3NSA, 5NSA, and 2,4DNP, with IC_50_ values of 90.8, 96.9, and 108.1 mg/L, respectively. For Oatp1d1, 5NSA and 2,4DNP showed the strongest inhibition, with IC_50_ values of 2.6 and 3.1 mg/L, respectively. The IC_50_ values for all tested NMAHs, with the exception of 4NG, were consistently lower for the Oatp1d1 transporter than for Oct1, suggesting a greater interaction potential toward Oatp1d1. This differential interaction may reflect compound-specific physicochemical properties, such as acidity (proton-donating ability) and associated polar surface area. Furthermore, mixtures of Σ_10_NMAHs and Σ_5_NMAHs (Supplementary Table S5) exhibited stronger interaction with Oatp1d1 compared to Oct1 (Figure 1C, and Supplementary Figure S8), underscoring the prominent role of 4NS and methylated NCs, the most abundant constituents in both mixtures. These findings point to selective transporter interactions by NMAHs that may influence their cellular uptake and toxicokinetic behavior in aquatic organisms.

The EROD bioassay revealed that neither the model NMAHs nor their mixtures were potent CYP1A1 inducers (Supplementary Tables S6 and S7, and Figures S9 and S10).

#### 3.2.3. Acute and Chronic Effects of NMAHs

The results of acute and chronic toxicity of the model NMAHs and their mixtures (Σ_10_NMAHs and Σ_5_NMAHs) are summarized in Figure 1D, and presented in more details in Supplementary Tables S8–S10 and Figures S11–S13. The strongest cytotoxic effects in PLHC-1 cells were induced by 4NS (IC_50_ = 2.0 mg/L), followed by NCs (IC_50_ = 3.1–4.1 mg/L). Other NMAHs showed no cytotoxicity at the tested concentrations. All compounds exhibited chronic toxicity toward algae, with NCs again showing the highest potency (IC_50_ = 0.6–1.1 mg/L), followed by 4NS (13.4 mg/L). Nitrophenols and NSAs were less toxic, with IC_50_ values ranging from 18.6 to 54.1 mg/L. Both NMAH mixtures induced concentration-dependent cytotoxic and algal inhibitory effects (1.0–25.8 mg/L), with nearly identical IC_50_ values. These results indicate that the most abundant compounds in the sBB_H2O_ extract, 4NC, methylated nitrocatechols, and 4NS, were the primary contributors to the observed toxicity.

#### 3.2.4. NMAHs Toxicity to Zebrafish Embryos

**Figure 3:**
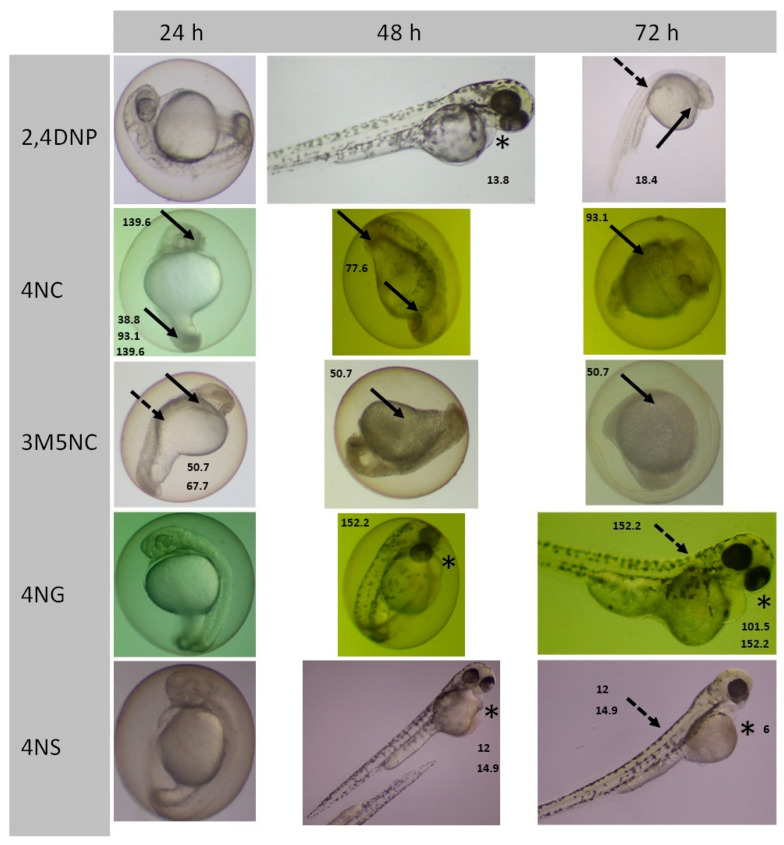
Representative morphological abnormalities observed in *Zebrafish* (*Danio rerio*) embryos following exposure to selected sublethal concentrations of 2,4DNP, 4NC, 3M5NC, 4NG, 4NS (indicated in mg/L). Noted deformations include cardiac edema (asterisk), spinal curvature (dashed arrow), and necrosis of head and tail (solid arrow).

Embryotoxicity of selected model NMAHs was evaluated in *Danio rerio* embryos using representatives of distinct structural classes: nitrophenols (2,4DNP), methoxynitrophenols (4NG, 4NS), nitrocatechols (4NC), and methylated nitrocatechols (3M5NC). Exposure to these compounds (4.6–203.0 mg/L) induced a range of morphological abnormalities, with compound-specific patterns (Table 2). The strongest embryotoxic response was observed for 4NS, which caused pericardial edema at 6.0 mg/L after 72 h, and complete lethality at 29.9 mg/L within 24 h (Figure 3). 2,4DNP was the second most potent, inducing hypopigmentation after 24 h and developmental delays after 48 h at concentrations of 13.8 and 9.2 mg/L, respectively. Severe malformations and lethality were recorded at 18.4 mg/L after 72 h. The remaining compounds followed the order of decreasing toxicity: 4NC > 3M5NC > 4NG, indicating structural influences on developmental outcomes.

#### 3.2.5. NMAHs Exposure Altered Lipids of Zebrafish Embryos

**Figure 4:**
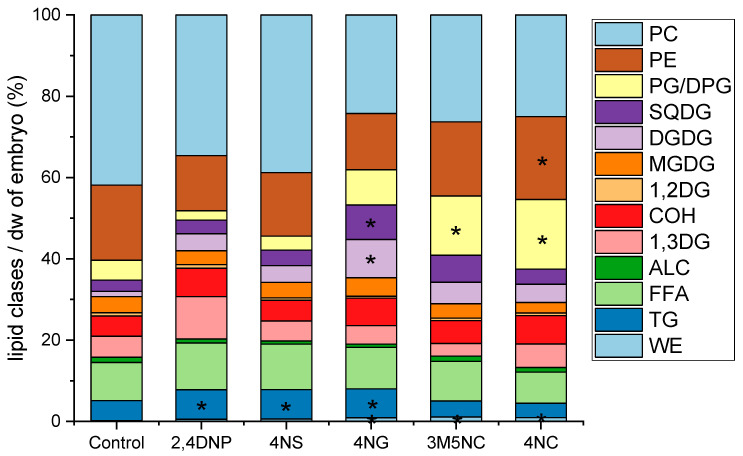
Lipid class composition (percent of dry weight) in zebrafish (*Danio rerio*) embryos at 48 h post-fertilization (hpf), unexposed (Control) and exposed to selected nitrated monoaromatic hydrocarbons (NMAHs): 2,4DNP (9.2 mg/L), 4NS (5.0 mg/L), 4NG (42.3 mg/L), 3M5NC (42.3 mg/L), and 4NC (38.8 mg/L). Lipid classes included: free fatty acids (FFA), triacylglycerols (TG), alcohols (ALC), cholesterol (COH), 1,2-diacylglycerols (1,2DG), 1,3-diacylglycerols (1,3DG), wax esters (WE), glycolipids (GL; monogalactosyldiacylglycerols, MGDG; digalactosyldiacylglycerols, DGDG; sulfoquinovosyldiacylglycerols, SQDG), and phospholipids (PL; phosphatidylglycerol, PG; diphosphatidylglycerol (DPG); phosphatidylethanolamine, PE; phosphatidylcholine, PC). The results represent mean of two exposure sets. Asterisks indicate a statistically significant increase (*p* < 0.05) in the percentage of a given lipid class relative to the control.

The total analysed lipid content of zebrafish embryos exposed to NMAHs for 48 hpf ranged from 23.2% (4NG) to 28.9% (4NC) of dry weight, representing a statistically significant increase compared to unexposed control (18.7%, *t*-test, *p* < 0.05). Triacylglycerol levels were significantly elevated in embryos exposed to 2,4DNP, 4NS, and 4NG, but not in those treated with 3M5NC or 4NC. Increases were also observed for PE in embryos exposed to 4NC; WE in those treated with 3M5NC, 4NC, and 4NG; and PG/DPG in those exposed to 3M5NC and 4NC. Additionally, DGDG and SQDG levels were significantly elevated in embryos exposed to 4NG (Figure 4).

## 4. Discussion

### 4.1. NMAHs Contributed to Ecotoxicity of Atmospheric Aerosols

The chemical composition of atmospheric aerosols governs their ecotoxicity [5]. A useful approach for characterizing toxic organic constituents involves comparing the biological effects of solvent-specific extracts from a single aerosol sample [49]. In this study, we extracted sBB aerosols using water, MeOH, DCM and Hex. Extracts were first assessed for their interaction with zebrafish membrane transporters involved in xenobiotic uptake (phase 0 of detoxification). All extracts showed activity, with sBB_H2O_ and sBB_MeOH_ exhibiting the strongest effects (Figure 1), consistent with a higher abundance of polar organic molecules. Quantification of individual NMAHs in sBB extracts confirmed their predominance in the H_2_O and MeOH fractions (Figure 2). Transporter assays further showed that sBB and AA aerosol extracts, mixtures of Σ_10_NMAHs and Σ_5_NMAHs, and individual NMAHs (excluding 4NG) more strongly interacted with anion transporters than cationic ones (Figure 1). Despite representing a small mass fraction (0.073% in sBB_H2O_, 0.24% in AA_H2O_), NMAHs likely contributed to the observed interactions of aerosols with membrane uptake transporters.

To assess toxicity, we performed MTT and algal growth inhibition assays. All sBB extracts reduced PLHC-1 fish cell viability, though with relatively high IC_50_ values (95.9–148.0 mg/L). In contrast, only a subset of individual NMAHs, 4NS, 4NC, and methyl nitrocatechols, exhibited acute cytotoxicity (IC_50_ = 2.0–4.1 mg/L; Figure 1). The Σ_10_NMAHs and Σ_5_NMAHs mixtures showed comparable toxicity across both assays, with IC_50_ of ~5–6 mg/L (Figure S13), suggesting these five NMAHs were primary contributors to the acute and chronic toxicity of sBB_H2O_ (Figure 1C), as in water extract were most abundant (Figure 2). In algae, however, the effects diverged. While sBB_H2O_ extract showed chronic toxicity, sBB_MeOH_, sBB_Hex_, sBB_DCM_, and AA_H2O_ extracts exhibited growth-promoting effects at lower concentrations, likely due to co-extracted nutrients (Supplementary Figure S5). Algal growth stimulation has been shown to mask toxicity in similar contexts [50]. The sBB_MeOH_ and sBB_DCM_ became inhibitory only above 800 and 220 mg/L, respectively. The AA_H2O_ did not show cytotoxicity within the tested range but may do so at higher PM concentrations (>50 mg/L). Further interpretation of these opposing effects would require a complete chemical profile of the aerosol extracts.

### 4.2. Interaction of NMAHs with Oct1 and Oatp1d1 Membrane Transporters

Screening assays revealed that the membrane transporters Oct1 and Oatp1d1 exhibit broad substrate specificity, consistent with previous findings demonstrating their ability to interact with a diverse array of endogenous compounds and environmentally relevant xenobiotics [30,51]. These transporters are highly expressed in toxicologically critical organs such as the liver and kidneys, underscoring their physiological relevance. In this study, we observed significantly stronger interactions between aerosol extracts and individual NMAHs with Oatp1d1 compared to Oct1. This pattern likely reflects differences in molecular charge and structural characteristics of the NMAHs, as well as the presence of other organic compounds in the aerosols. The ionization states of the tested NMAHs under experimental conditions provide a mechanistic explanation for this trend (Table 1). Specifically, NMAHs are expected to exist predominantly in their deprotonated (anionic) forms at the pH used in the assays. This negative charge favours interactions with anion transporters such as Oatp1d1. Moreover, the presence of one or more nitro groups on the aromatic ring increases the electron–withdrawing capacity of the molecule, enhancing both its acidity and its potential to interact with charged transporter binding sites. These electronic effects may not only facilitate ionization but also contribute to stronger binding affinities with anion-specific membrane proteins, further supporting the observed transporter preference.

To further explore structure-activity relationships, we examined the correlation between the topological polar surface area of NMAHs and their IC_50_ values for Oct1 and Oatp1d1. Topological polar surface area, defined as the sum of the surface area of all polar atoms (primarily oxygen and nitrogen, including attached hydrogens), serves as a proxy for molecular polarity [52]. Statistically significant correlations were observed (*p* < 0.05), with a strong positive correlation for Oct1 (r = 0.737) and a strong negative correlation for Oatp1d1 (r = −0.697). These opposing trends likely reflect structural differences between the two transporters. Oatp1d1, which contains a single binding site [53], may favour more polar molecules due to favourable electrostatic or hydrogen-bonding interactions. In contrast, Oct1 features multiple binding regions [30], where high polarity may hinder molecular alignment or access, reducing interaction efficiency. Overall, these findings suggest that NMAH transport is governed by both molecular polarity and the structural characteristics of the transporter, influencing their cellular uptake and potential toxicity.

### 4.3. Role of Aerosols and NMAHs in Phase I of Cellular Detoxification

A key mechanism in cellular detoxification involves the biotransformation of xenobiotics into more hydrophilic forms, primarily mediated by cytochrome P450 enzymes (CYPs). Within this enzyme family, the CYP1 group, especially CYP1A1, plays a central role in phase I detoxification in teleost fish. To assess whether NMAHs could induce CYP1A1 activity, we performed the EROD bioassay using both individual NMAHs and sBB and AA extracts. Only AA_H2O_ showed a clear inductive effect (Supplementary Figures S3, S9 and S10). To explain this discrepancy, we quantified 11 priority polycyclic aromatic hydrocarbons (PAHs) in both aerosol types (described in Supplementary Materials), as PAHs are well-established inducers of CYP1A1 at both the transcript and enzymatic activity levels [54,55,56]. Although PAHs accounted for just 1.2% of sBB mass, they made up 15.5% of AA mass (Supplementary Table S2), aligning with the observed phase I response of the AA extract (EC_50_ = 31.8 mg/L, Supplementary Figure S3). Notably, PAHs were originally extracted using a cyclohexane/toluene mixture, which ensured high recovery (details in Supplementary Materials). However, for our bioassays, we employed sequential extractions with H_2_O, MeOH, Hex, and DCM, an approach less efficient for hydrophobic compounds like PAHs [49]. As a result, the sBB extracts likely contained negligible amounts of PAHs, explaining the lack of CYP1A1 induction, while the AA_H2O_ had sufficient PAHs to elicit a modest but detectable enzymatic response.

### 4.4. NMAHs Impacted Lipid Homeostasis of Zebrafish Embryos

Lipids serve critical roles during zebrafish embryogenesis, supporting energy demands and cellular structure. At early developmental stages, cholesterol, phospholipids, and triacylglycerols are predominant, comprising ~40%, 35%, and 9% of total lipids, respectively [41]. Sublethal exposure to slightly lipophilic compounds like 2,4DNP alters lipid profiles by incorporating into membranes and increasing fluidity, which triggers a compensatory “homeoviscous adaptation” involving a shift to more saturated phospholipid chains [40]. In this study, zebrafish embryos exposed for 48 hpf to 2,4DNP (9.2 mg/L), 4NS (5.0 mg/L), 4NG (42.3 mg/L), 3M5NC (42.3 mg/L), and 4NC (38.8 mg/L) exhibited statistically significant increases in triacylglycerols, phospholipids, glycolipids, and wax esters, indicating disrupted lipid homeostasis. The degree of disruption followed the order: 4NG > 4NC > 3M5NC > 2,4DNP ≈ 4NS. These changes reflect compound-specific impacts on lipid metabolism and membrane integrity. Given that the Fish Embryo Toxicity test is predictive of adult responses [57], similar lipid alterations likely occur in mature zebrafish. Consistent with this, adult zebrafish exposed to 2,4DNP under physical stress show increased triacylglycerols accumulation [58]. Together, these findings underscore the sublethal yet physiologically significant effects of atmospheric NMAHs on lipid regulation in aquatic organisms.

### 4.5. Ecological Impact of NMAHs Under Climate Changes

The *in vitro* and *in vivo* ecotoxicological analysis of model NMAHs and their relevant water mixtures (Σ_10_NMAHs and Σ_5_NMAHs) revealed IC_50_ values in the mg/L range. Specifically, the IC_50_ for interaction of NMAH with transmembrane proteins was in the range 2.6 mg/L (5NSA, Oatp1d1)–108.1 mg/L (2,4DNP, Oct1). For acute toxicity to fish cells, IC_50_ values were 2.0 mg/L (4NS)–4.1 mg/L (3M5NC), while the chronic toxicity to algae showed IC_50_ from 0.6 to 1.1 mg/L (NCs) and 13.4 to 54.1 mg/L (NPs, NSAs). These values are consistent with the toxic values for some NMAHs reported for other aquatic organisms. For example, the obtained EC_50_ values for the toxicity of guaiacol (GUA), 6-nitroguaiacol (6NG), 4NG, and 4,6-dinitroguaiacol (4,6DNG) to marine luminescent bacteria *Vibrio fisheri* ranged from 16.7 to 102 mg/L [20]. Furthermore, organisms at higher levels of biological organization also exhibit negative effects upon exposure to NMAHs. In our experiments, zebrafish embryos showed significant negative developmental effects (Table 2, Figure 3), accompanied by reduced biomass and altered composition of lipids (Figure 4). The LC_50_ (half lethal concentration) values of 18.7 mg/L and 9.7 mg/L and EC_50_ values of 7.9 mg/L and 3.1 mg/L were obtained after 48 h exposure of zebrafish embryos to 2NP and 2,4DNP, respectively [21], similar to the recently reported values for NCs [23]. Moreover, a significant reduction in growth and biomass and poor photochemical efficiency of photosystem II of terrestrial plants were observed during a two-week long root exposure to 4NG [59].

In recent decades, climate actions related to the Paris Agreement and rising fuel prices are likely to increase the use of biomass as an energy source [60]. Concurrently, global warming and droughts associated with climate changes [61] have increased the frequency and scale of forest fires, amplifying BB emissions worldwide. These trends suggest a likely rise in airborne concentrations of wood smoke and its associated pollutants, including NMAHs. Therefore, although NMAH concentrations in surface waters [18,62,63] appear lower than the IC_50_ values obtained in our bioassays, early negative effects of NMAHs exposure could become more significant. Furthermore, predictions indicate that PM_2.5_ levels in heavily polluted anthropogenic regions may not decline significantly [64]. Both these intensify concerns over aerosol deposition rate into near aquatic systems and accompanied ecotoxic effects.

## 5. Conclusions

Our study provides new insights into the environmental hazards associated with deposition of polluted atmospheric aerosols through examination of their chemical composition, particularly NMAHs and their ecotoxicity. Using a suite of *in vivo* and *in vitro* bioassays, we established a direct link between the toxicity of source-specific sBB and AA aerosol extracts and their NMAH content. Among the tested compounds, the most abundant NMAHs (3M5NC, 4M5NC, 3M4NC, 4NC, and 4NS in sBB; 4NC, 3M5NC, 4M5NC, and 3M4NC in AA) exhibited toxic effects on aquatic organisms in mg/L concentration range. Mixtures of these NMAHs were shown to drive the acute toxicity of sBB to fish cells and the chronic toxicity to algae, mirroring the toxic effects of individual NMAHs. Furthermore, 4NS emerged as the most disruptive to zebrafish embryonic development, followed by 2,4DNP > 4NC > 3M5NC > 4NG, while the sublethal NMAHs concentrations altered lipid homeostasis in embryos in the order 4NG >4NC > 3M5NC > 2,4DNP, 4NS. These findings emphasize the pressing need for continued monitoring of atmospheric particle-bound NMAHs, particularly NCs, as emerging ecotoxic BB tracers. Their increasing presence in atmospheric and aquatic compartments demands further toxicological evaluations across multiple trophic levels, alongside biochemical studies, to comprehensively assess their impact on environmental and public health. Addressing these challenges will be critical for mitigating the growing ecological risks especially associated with climate-driven BB emissions.

## Figures and Tables

**Table 1:** Targeted atmospheric NMAHs, their structures, molecular weights (MW), octanol–water partitioning coefficients (K_ow_) (EPI SuiteTM v4.11, U. S. EPA (2023)), topological polar surface areas (TPSA [43], computed by Cactvs 3.4.8.18 (PubChem release 2021.05.07), National Library of Medicine), and dissociation constants (p*K*_i_). nd—no data found. /—no p*K*_2_.

NMAH	Structure	MW	log *K*_ow_^a^	TPSA ^b^ /Å^2^	p*K*_1_	p*K*_2_
4-nitrophenol (4NP)	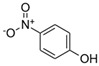	139.11	1.91	66	7.1 ^c^	/
2-methyl-4-nitrophenol (2M4NP)	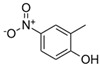	153.14	2.46	66	nd	/
3-methyl-4-nitrophenol (3M4NP)	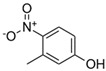	153.14	2.46	66	7.33 ^d^	/
2,4-dinitrophenol (2,4DNP)	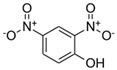	184.11	1.7	112	4.1 ^e^	/
4-nitrosyringol (4NS)	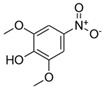	199.16	nd	84.5	nd	/
4-nitroguaiacol (4NG)	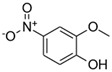	169.13	1.73	75.3	nd	/
4-nitrocatechol (4NC)	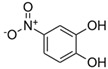	155.11	1.66	86.3	6.6 ^f^	10.8 ^f^
3-methyl-4-nitrocatechol (3M4NC)	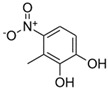	169.13	nd	86.3	nd	nd
3-methyl-5-nitrocatechol (3M5NC)	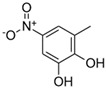	169.13	2.14	86.3	nd	nd
4-methyl-5-nitrocatechol (4M5NC)	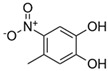	169.13	1.98	86.3	nd	nd
5-nitrosalicylic acid (5NSA)	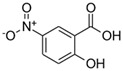	183.12	2.64	103	8.9 ^f^	10.9 ^f^
3-nitrosalicylic acid (3NSA)	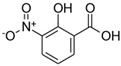	183.12	2.64	103	nd	nd

^a^ EPI SuiteTM v4.11, U. S. EPA (2023). ^b^ Computed by Cactvs 3.4.8.18 (PubChem release 2021.05.07), National Library of Medicine. ^c^ [44]. ^d^ [45]. ^e^ [46]. ^f^ [47].

**Table 2:** Nitrated monoaromatic hydrocarbons (NMAHs) and their effects on embryonic development and survival in *Danio rerio*. Check marks (✓) indicate concentrations at which no developmental abnormalities were observed. Concentrations used in sublethal lipid homeostasis assays are denoted with an asterisk (*).

NMAH	Concentration (mg/L)	Observed Effects		
		24 h	48 h	72 h
2,4-dinitrophenol (2,4DNP)	4.6	✓	✓	✓
	9.2 *	✓	Delayed development	Delayed development
	13.8	Weak or no pigmentation	Irregular yolk shape Heart edema	Irregular yolk shape
	18.4	Weak or no pigmentation	Spine malformations	Spine and lethal malformations
4-nitrocatechol (4NC)	7.8	✓	✓	✓
	15.5	✓	Delayed development	✓
	38.8 *	Head malformations Tail necrosis Weak or no pigmentation	Delayed development	✓
	77.6	✓	Delayed development Tail necrosis Weak or no pigmentation	Spine malformations
	93.1	Heart edema Tail necrosis Weak or no pigmentation	Spine malformations	Necrosis
	139.6	Head and tail necrosis	Lethal malformations	Lethal malformations
	186.1	Lethal malformations	Lethal malformations	Lethal malformations
3-methyl-5nitrocatechols (3M5NC)	8.5	✓	✓	✓
	16.9	✓	✓	✓
	42.3 *	✓	✓	✓
	50.7	Head and tail necrosis Irregular yolk shape	Delayed development Necrosis	Lethal malformations
	67.7	Head and tail necrosis	Lethal malformations	Lethal malformations
	84.6	Lethal malformations	Lethal malformations	Lethal malformations
	101.5	Lethal malformations	Lethal malformations	Lethal malformations
	152.2	Lethal malformations	Lethal malformations	
4-nitroguaiacol (4NG)	8.5	✓	Delayed development	✓
	16.9	✓	Delayed development	✓
	42.3 *	Weak or no pigmentation	Delayed development	✓
	84.6	Head and tail necrosis Lethal malformations	Delayed development	✓
	101.5	Necrosis	Delayed development	Delayed development Heart edema
	152.2	Necrosis	Delayed development Heart edema	Heart edema Spine malformations
	203.0	Lethal malformations	Delayed development Heart edema	Malformations
4-nitrosyringol (4NS)	5.0 *	✓	✓	✓
	6.0	✓	✓	Heart edema
	10.0	✓	✓	Heart edema
	12.0	Necrosis	Heart edema	Necrosis Spine malformations
	14.9	Necrosis	Heart edema	Necrosis Spine malformations
	29.9	Lethal Malformations	Lethal malformations	Lethal malformations
	44.8	Lethal Malformations	Lethal malformations	Lethal malformations
	59.8	Lethal Malformations	Lethal malformations	Lethal malformations

## Data Availability

The original contributions presented in this study are included in the article/supplementary material. Further inquiries can be directed to the corresponding authors.

## Supplementary Materials

The following supporting information can be downloaded at: https://www.mdpi.com/article/10.3390/toxics13121037/s1, Table S1: The range of concentrations (mg/L) of sBB and AA aerosols applied in bioassays with phase 0 protein transporters of cellular detoxification, and in MTT, AlgaeTox and EROD bioassay; Figure S1: *In vitro* determination of the inhibition of the transport activity of the zebrafish organic cation transporte-r Oct1 stably expressed in the cell line Flp-In-293-drOct1 by measuring the inhibition of the uptake of the model substrate ASP+ (%) after incubation with a range of concentrations of sBB and AA extracts; Figure S2: *In vitro* determination of the inhibition of the transport activity of the zebrafish organic anion transporter Oatp1d1 stably expressed in the cell line Flp-In-293-drOatp1d1 by measuring the inhibition of the uptake of the model substrate LY (%) after incubation with a range of concentrations of sBB and AA extracts; Figure S3: Determination of phase I detoxification enzyme (CYP1A1) induction in PLHC-1 cells by the EROD bioassay after exposure to (A) sBB and (B) AA aerosol extracts; Figure S4: *In vitro* determination of the acute cytotoxic effect of sBB and AA extracts using the MTT assay after 72 h of sample exposure; Figure S5: *In vivo* determination of chronic toxic effects of sBB and AA extracts by the AlgaeTox test on the unicellular green freshwater alga *Scenedesmus subspicatus* after 96 h of exposure; Table S2: PAH concentrations determined in sBB and AA aerosols; Table S3: Model NMAHs concentrations prepared for *in vitro* determination of the inhibition of the transport activity of the zebrafish Oct1 organic cation transporter; Figure S6: Dose-response curves of the *in vitro* inhibition of the transport activity of the zebrafish organic cation transporter Oct1 stably expressed in the cell lines Flp-In-293-drOct1 by measuring the inhibition of the uptake of the model substrate ASP+ (%), after incubation with a series of model NMAHs concentrations; Table S4: Model NMAHs concentrations prepared for *in vitro* determination of the inhibition of the transport activity of the zebrafish Oatp1d1 organic anions transporter; Figure S7: Dose-response curves of the *in vitro* inhibition of the transport activity of the zebrafish organic anion transporter Oatp1d1 stably expressed in the cell lines Flp-In-293-drOatp1d1 by measuring the inhibition of the uptake of the model substrate and LY (%) after incubation with a series of model NMAHs concentrations; Table S5: Concentrations of NMAHs mixtures prepared for *in vitro* determination of the inhibition of the transport activity of the zebrafish Oatp1d1 organic anions transporter and Oct1 organic cation transporter; Figure S8: Dose-response curves of the *in vitro* inhibition of the transport activity of the zebrafish organic cation transporter Oct1 (A) and organic anion transporter Oatp1d1 (B) after incubation with a NMAHs mixtures (10 NMAHs and 5 NMAHs) concentrations; Table S6: Model NMAHs concentrations prepared for *in vitro* determination of induction potential of CYP1A1 detoxification enzymes by measuring the ethoxyresorufin-O-deethylase (EROD) activity in PLHC-1 fish cells; Figure S9: EROD bioassay for model 2,3,7,8-tetrachlorodibenzo-p-dioxin (TCDD) and tested NMAHs; Table S7. Concentrations of model NMAHs mixtures for *in vitro* determination of induction potential of CYP1A1 detoxification enzymes by the EROD bioassay; Figure S10: EROD bioassay for the mixture of 10 NMAHs and 5 NMAHs; Table S8: Model NMAHs concentrations prepared for *in vitro* determination of the acute cytotoxic effect on fish PLHC-1 cells by the MTT assay; Figure S11: *In vitro* determination of the acute cytotoxic effect of individual model NMAHs by the MTT assay after exposure to NMAHs concentrations for 72 h; Table S9: Model NMAHs concentrations prepared for *in vivo* determination of chronic toxic effects on freshwater green algae *Scenedesmus subspicatus* by the AlgaeTox assay; Figure S12: Results of *in vivo* determination of chronic toxic effects of individual model NMAHs by the AlgaeTox test after 96-h exposure to a range of NMAHs concentrations; Table S10: Concentrations of model NMAHs mixtures prepared for *in vitro* determination of acute and *in vivo* determination of chronic toxic effects by the MTT and AlgaeTox test, respectively; Figure S13. Results of determination of acute (A) and chronic (B) toxic effects of NMAHs mixtures (10 NMAHs and 5 NMAHs) by the MTT test and AlgaeTox test, respectively (References [65,66,67,68] are cited in the Supplementary Materials).

## Abbreviations

The following abbreviations are used in this manuscript:

1,2DG	1,2-Diacylglycerols
1,3DG	1,3-Diacylglycerols
AA	Ambient anthropogenic
AA_H2O_	Extract of AA aerosols in water
ALC	Alcohols
ASP+	4-(4-(dimethylamino)styryl)-N-methylpyridinium iodide
BB	Biomass burning
COH	Cholesterol
DCM	Dichloromethane
DGDG	Digalactosyldiacylglycerols
EC_50_	Half-maximal effect concentration of xenobiotic
FFA	Free Fatty Acids
GL	Glycolipids
GUA	Guaiacol
Hex	Hexane
LC_50_	Half-maximal inhibitory concentration
LC-MS/MS	Liquid chromatography-mass spectrometry/mass spectrometry
LY	Lucifer yellow
MeOH	Methanol
MGDG	Monogalactosyldiacylglycerols
MTT	3-(4,5-dimethyl-2-thiazolyl)-2,5-diphenyl-2H-tetrazolium bromide
NC	Nitrocatechol
NG	nitroguaiacol
NMAH	Nitrated monoaromatic hydrocarbons
NP	Nitrophenol
NS	Nitrosyringol
NSA	Nitrosalicylic acid
Oatp1d1	Organic anion-transporting polypeptide 1d1
Oct1	Organic cation transporter 1
PAHs	Polycyclic aromatic hydrocarbons
PC	Phosphatidylcholine
PE	Phosphatidylethanolamine
PG	Phosphatidylglycerol
PL	Phospholipids
sBB	Simulated biomass burning
sBB_DCM_	Extract of sBB aerosols in dichloromethane
sBB_H2O_	Extract of sBB aerosols in water
sBB_Hex_	Extract of sBB aerosols in hexane
sBB_MeOH_	Extract of sBB aerosols in methanol
SQDG	Sulfoquinovosyldiacylglycerols
TG	Triacylglycerols
TPSA	Topological polar surface area
WE	Wax esters
