# Supplementary material for: Ecotoxicity of Nitrated Monoaromatic Hydrocarbons in Aquatic Systems: Emerging Risks from Atmospheric Deposition of Biomass Burning and Anthropogenic Aerosols
Source: Toxics. 2025 Nov 30;13(12):1037. doi: 10.3390/toxics13121037 (PMC12737277; doi:10.3390/toxics13121037)
Supplement: Supplementary file 1 [file toxics-13-01037-s001.zip › toxics-3990674-supplementary.pdf]

Supplementary Materials: Ecotoxicity of Nitrated Monoaromatic Hydrocarbons in Aquatic Systems: Emerging Risks from Atmospheric Deposition of Biomass Burning and Anthropogenic Aerosols

Saranda Bakija Alempijević [1], Slađana Strmečki [1,*], Ivan Mihaljević [1,*], Sanja Frka [1], Jelena Dragojević [1], Ivana Jakovljević [2] and Tvrtko Smital [1]

[1] Division for Marine and Environmental Research, Ruđer Bošković Institute, Bijenička 54, 10 000 Zagreb, Croatia; saranda.bakija.alempijevic@irb.hr (S.B.A.); frka@irb.hr (S.F.); jelena.dragojevic@irb.hr (J.D.); smital@irb.hr (T.S.)
[2] Institute for Medical Research and Occupational Health, Ksaverska Cesta 2, 10 000 Zagreb, Croatia; ijakovljevic@imi.hr
* Correspondence: strmecki@irb.hr (S.S.); ivan.mihaljevic@irb.hr (I.M.)

Experimental

Oct1 and Oatp1d1

For both assays, cells were grown in DMEM-FBS media, which was Dulbecco's modified Eagle medium (DMEM) with high glucose content (Capricorn Scientific, Ebsdorfergrund, Germany) and 10% fetal bovine serum (FBS) (Capricorn Scientific, Ebsdorfergrund, Germany), maintained at 37 °C and 5% CO_2. 24 hours before the experiment, cells were seeded in 96-well plates at a density of $7x10^5$ or $8x10^5$ cells/mL, with a final volume of 125 μL per well. The assays were performed when confluency of the cells was above 90%.

In the Oatp1d1 assay, the medium was removed after the cells had been seeded for 24 hours, and the cells were pre-incubated with 75 μL of transport medium (145 mM NaCl, 3 mM KCl, 1 mM $CaCl_2$, 0.5 mM $MgCl_2$, 5 mM D-glucose, and 5 mM HEPES) for 10 minutes at 37°C. Then, 25 μL of the test substances (5-fold concentration) were added to the pre-incubation medium. After 40 s, 25 μL of the 5-fold concentrated fluorescent substrate LY was added and incubated for 15 min at 37°C. After incubation, cells were washed twice with 125 μL of cold transport medium before lysing in 0.1% sodium dodecyl sulphate (Carl Roth, Karlsruhe, Germany) for 25 min at 37°C. The lysed cells were transferred to black 96-well microplates and LY fluorescence at 425/540 nm was measured.

In the Oct1 assay, DMEM-FBS medium was also removed after the cells had been seeded for 24 hours, and the cells were pre-incubated with 75 μL of transport medium for 5 minutes at 37°C. After incubation, 25 μL of the test compound and then 25 μL of ASP+ were added to the wells. Finally, fluorescence of the ASP+ was measured at 450/590 nm (extracts) and 470/605 nm (test NMAHs).

The Oct1 kinetics tests revealed that, at certain higher concentrations of the test NMAHs (Table S3), the passive fluorescence value (performed at a wavelength of 450/590 nm and 1 μM of ASP+) decreased in the zero minute. We investigated several possible reasons for that. First, we found out that NMAHs do not fluoresce themselves at 450/590 nm. Then, we conducted the Oct1 endpoint test in accordance with the test protocol with the Oatp1d1 protein transporter at 450/590 nm, with a change in the incubation time to 5 min after the addition of dye and then with a change in the concentration of the ASP+ substrate to 5 μM in the final solution. Since no significant difference in fluorescence was obtained between the exposed cells transfected with the Oct1 protein and Mock cells we extended the incubation time to 10 min and the concentration of ASP+ to 20 μM. After the test was carried out under the specified conditions, the cells in the wells were washed out. Then, using 250 μL instead of the 125 μL used in the earlier tests, we carried out an endpoint test in 48-well

plates. The test was run at 24 and 48 hours, with 20 μM of ASP+ and a 10-minute incubation period following dye addition. The results obtained were also not good.
Then, we used a medium without cells with the Oct1 protein. In the first experiment, the concentrations of the tested compounds varied (over a certain range), while the concentration of the ASP+ remained constant at 25 μM. In the second experiment, the concentration where we noticed a decrease of fluorescence at zero minute of a specific compound was fixed while adding ASP+ substrate over a specific range of concentrations. The measurements were performed at a wavelength of 470/605 nm. The calibration curves that were made corresponded to the calibration curves of the substrate ASP+ itself, indicating that the tested NMAHs do not react with ASP+. Furthermore, we carried out the test, again as a kinetic one, according to the protocol described in 2.4.1, but first adding the substrate to the cells, and then the tested compound. Such change was not beneficial for passive fluorescence. It is also possible that NMAHs at higher concentrations induce cellular membrane depolarization because they are composed of phenol or catechol for which it is known that lower passive cellular uptake [65, 66].
It is known that catechols at some experimental conditions can induce fluorescence quenching [67]. However, if fluorescence quenching happened during our experiments, obtained toxic effects are underestimated. Further experiments should be carried out to elucidate observed effect. Finally, we conducted tests at a wavelength of 470/605 nm and 0.5 μM ASP+ because under these conditions dose-response curves with fewer deviations were obtained. When conducting tests with aerosol extracts (Figure S1), we did not observe a decrease in fluorescence in the zero minute at higher concentrations and the wavelength of 450/590 nm and the concentration of ASP+ of 1 μM.

EROD test

Cells ($5x10^5$/mL) in a volume of 200 μL DMEM/F12 medium (Capricorn Scientific, Ebsdorfergrund, Germany) containing 5% FBS were inoculated into a 96-well microplate and incubated at 30 °C for 24 hours. Then, 100 μL of the medium was discarded and replaced with 100 μL of dissolved extracts or model NMAHs. After the 24-hour incubation at 30 °C, the medium over the cells was removed, the cells were washed twice with PBS, and 100 μL of 2 μM solution of ethoxyresorufin dissolved in phosphate buffer was added.

MTT assay

PLHC-1 fish cells were seeded at densities of $25x10^4$ cells/mL into 96-well microplate in DMEM-F12 medium (with 5% of FBS), with a final volume of 200 μL per well. After incubation for 24h at 30°C 100 μL of the medium from wells was discarded and added 100 μL of dissolved extracts or model NMAHs in concentration range which was two-fold concentrated. After 72h of incubation at 30°C the content above the cells was removed and cells were washed with 100 μL of PBS. Then, the fluid was removed and the cells were treated with 0.5 mg/ml MTT dye dissolved in DMEM/F12 for 3 hours. A yellow tetrazole is reduced to purple formazan salts, which were dissolved in isopropanol. The samples in the microplates were then shaked for 15 minutes at 350 rpm. The absorbance of the obtained solution was measured at 578 nm with reference filter on 750 nm.

Analysis of PM_{10}-bounded PAHs

Extraction of PAHs from aerosol samples was described in previous paper [68]. In brief, sBB and AA filters (see 2.2 main text) were extracted by ultrasonic with a solvent mixture (cyclohexane : toluene, 3 : 7), centrifuged and evaporated to dryness. They were then redissolved in acetonitrile. The analysis was performed using Agilent Infinity 1260 high-performance liquid chromatography (HPLC) with a fluorescence detector. Eclipse PAH stainless steel columns (100 × 4.6 mm), set to 20 °C, were used for PAH separations. The mobile phase was a mixture of water and acetonitrile (40:60) and the flow rate was 1 mL/min. The samples were analysed for the following PAHs: fluoranthene (Flu), pyrene (Pyr), benzo(a)anthracene (BaA), chrysene (Chry), benzo(b)fluoranthene (BbF), benzo(k)fluoranthene (BkF),

benzo(j)fluoranthene (BjF), benzo(a)pyrene (BaP), dibenzo(a,h)anthracene (DahA), benzo(ghi)perylene (BghiP), and indeno(1,2,3-cd)pyrene (IP). The method quantification limits (QL) were calculated as concentration equivalents to ten times the signal-to-noise ratio. The QL ranged from 0.002 ng/m^3 for BaA to 0.1 ng/m^3 for BjF, while the QL for BaP was 0.003 ng/m^3. The method accuracy extended between 88% for Flu to 109% for BkF.

Results

Table S1. The range of concentrations (mg/L) of sBB and AA aerosols applied in bioassays with phase 0 protein transporters of cellular detoxification mechanism (in total eight concentrations), and in MTT, AlgaeTox and EROD bioassay (in total six concentrations). Concentrations are calculated as particle mass found on the filter sections and diluted in appropriate volume of DMSO.

Bioassay		sBB_{H2O}	sBB_{MeOH}	sBB_{DCM}	sBB_{Hex}	AA_{H2O}
		mg/L				
phase 0 of cellular detoxification mechanism		2.8	4.6	13.8	13.8	0.4
		5.5	9.2	27.6	27.6	0.8
	EROD, MTT, AlgaeTox	11.0	18.4	55.2	55.2	1.6
		22.1	36.8	110.4	110.4	3.1
		44.1	73.6	220.8	220.8	6.3
		88.3	147.3	441.5	441.5	12.5
		176.5	294.5	883.0	883.0	25.0
		353.0	589.0	1766.0	1766.0	50.0

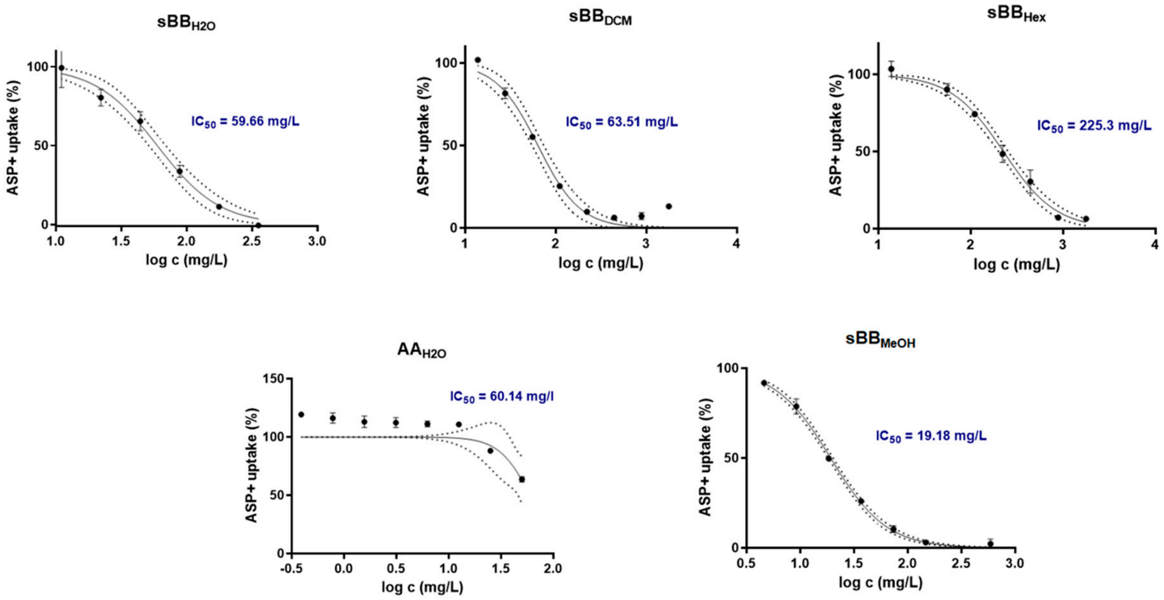

Figure S1. *In vitro* determination of the inhibition of the transport activity of the zebrafish organic cation transporter **Oct1** stably expressed in the cell line Flp-In-293-drOct1 by measuring the inhibition of the uptake of the model substrate ASP+ (%) after incubation with a range of concentrations of **sBB and AA extracts.**

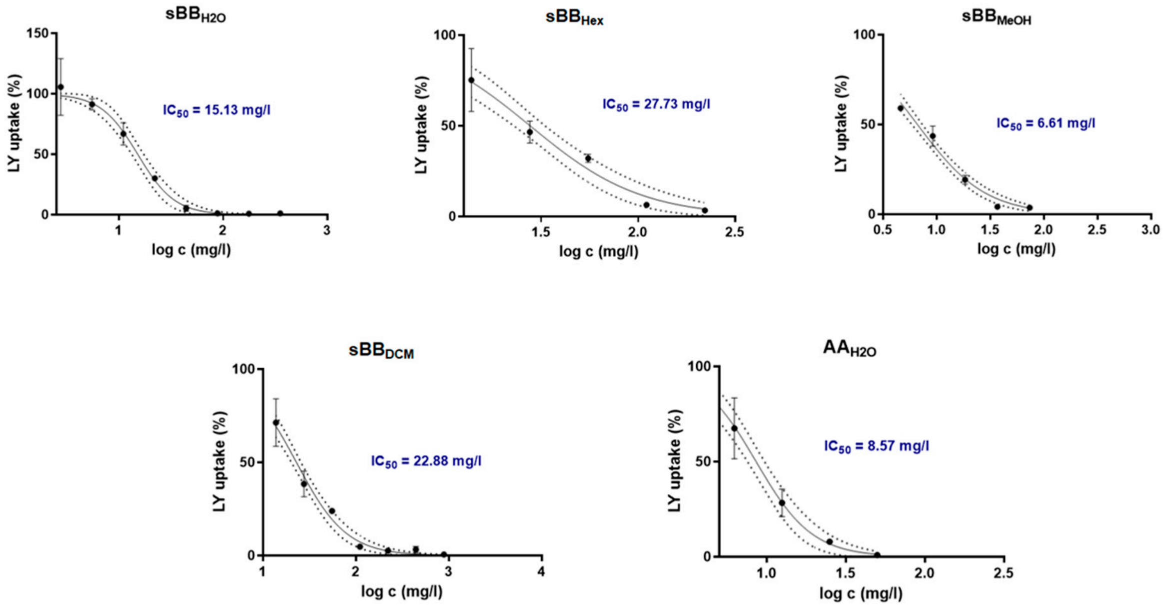

Figure S2. *In vitro* determination of the inhibition of the transport activity of the zebrafish organic anion transporter **Oatp1d1** stably expressed in the cell line Flp-In-293-drOatp1d1 by measuring the inhibition of the uptake of the model substrate LY (%) after incubation with a range of concentrations of **sBB and AA extracts.**

A

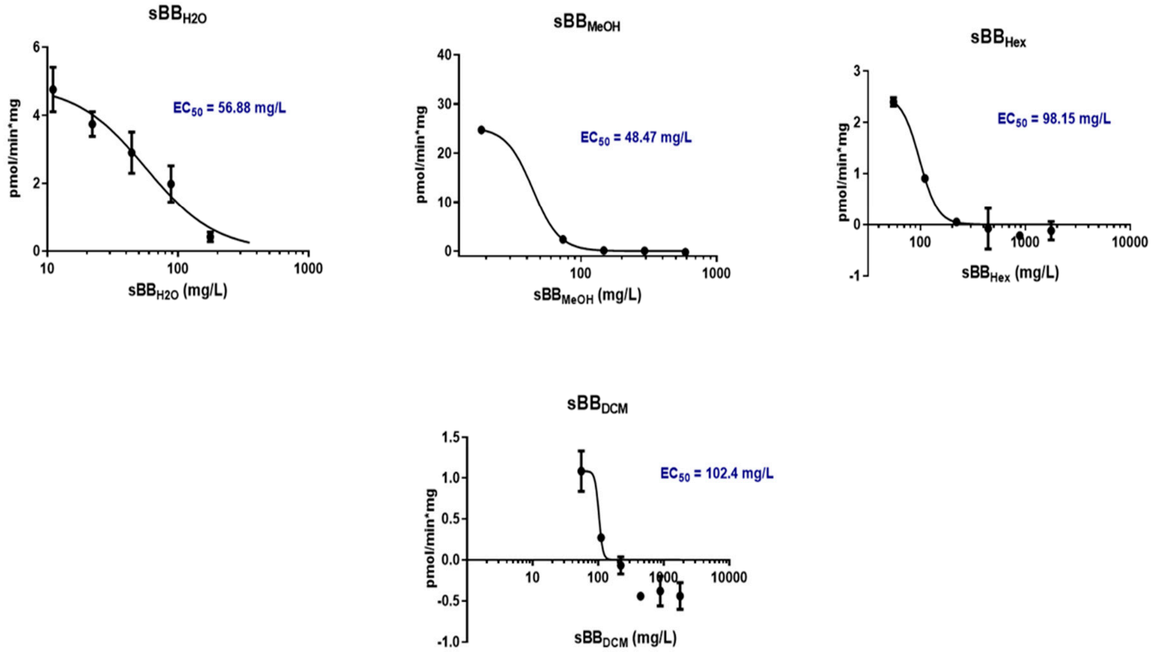

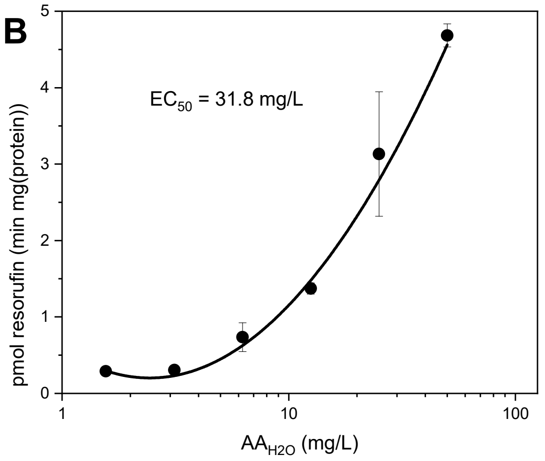

Figure S3. Determination of phase I detoxification enzyme (CYP1A1) induction in PLHC-1 cells by the **EROD** bioassay after exposure to **A) sBB and B) AA aerosol extracts**.

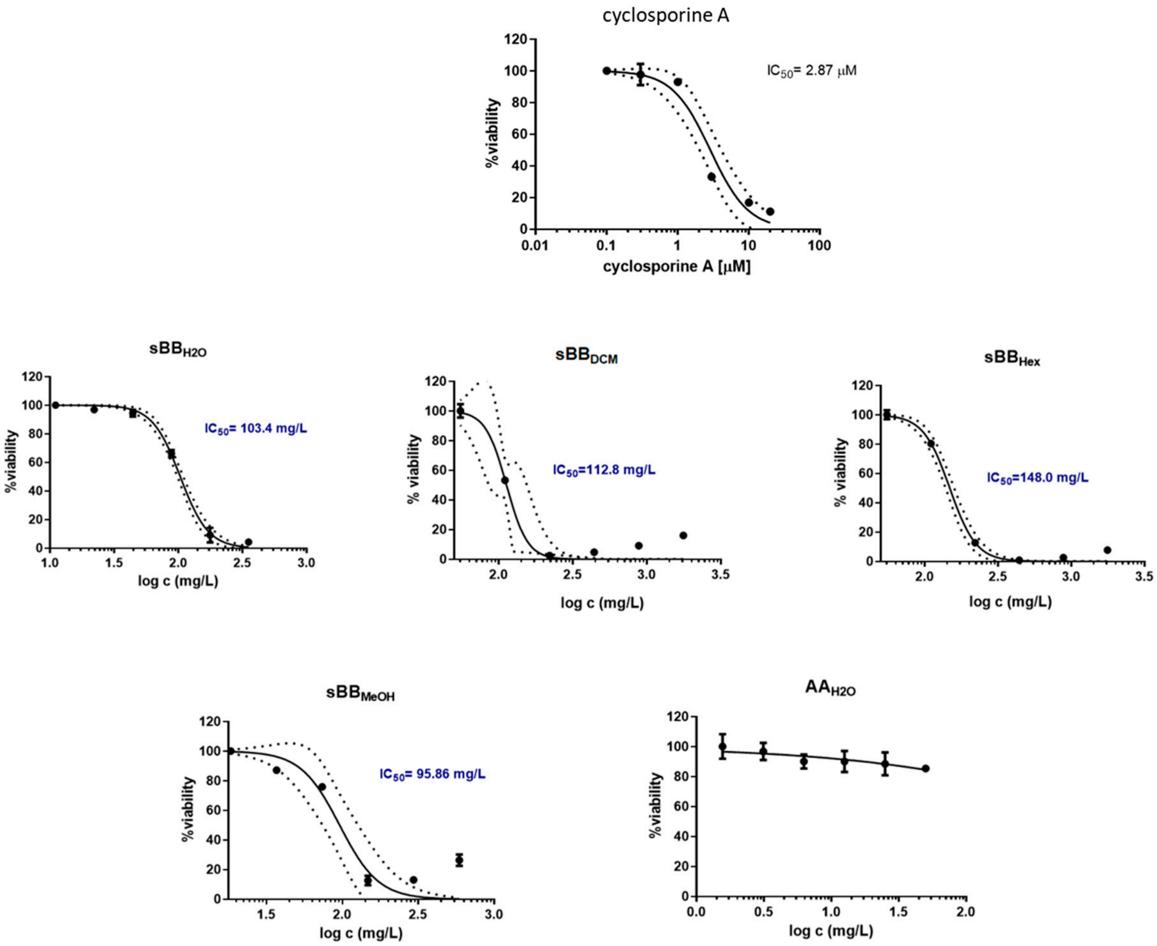

Figure S4. *In vitro* determination of the acute cytotoxic effect of **sBB and AA extracts** using the **MTT assay** after 72 hours of sample exposure.

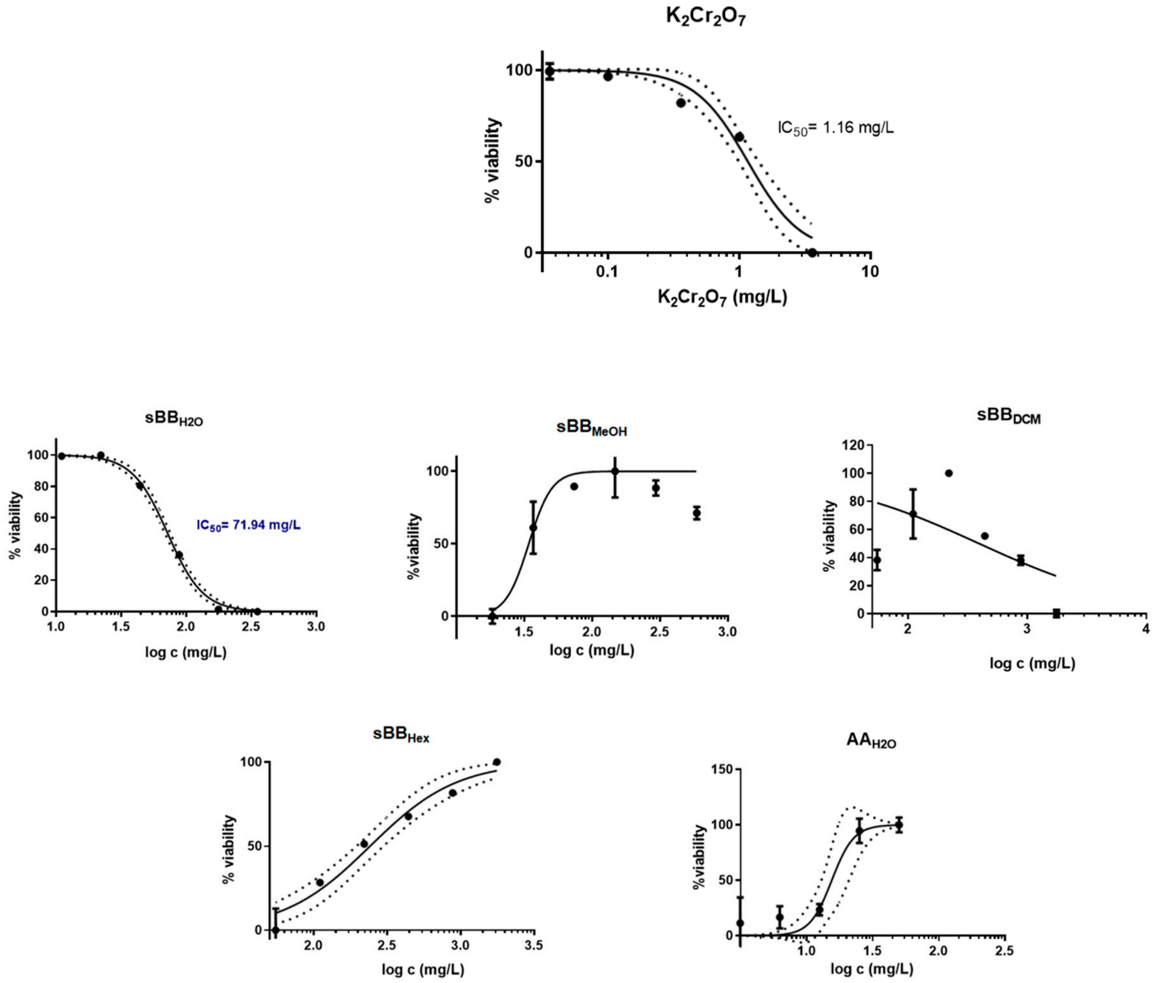

Figure S5. *In vivo* determination of chronic toxic effects of **sBB and AA extracts** by the **AlgaeTox test** on the unicellular green fresh-water alga *Scenedesmus subspicatus* after 96 hours of exposure.

Table S2. PAH concentrations determined in sBB and AA aerosols.

PAH	µg/mg sBB particles	µg in 17.66 mg of sBB	µg/mg AA particles	µg in 2.5 mg of AA
**BaP**	0.9	16.0	20.8	52.1
**Flu**	2.9	52.1	8.7	21.8
**Pyr**	3.3	58.6	8.2	20.4
**BjF**	0.4	6.9	12.0	30.0
**BbF**	0.8	13.8	23.3	58.2
**BkF**	0.3	4.8	8.8	22.0
**DahA**	0.1	1.2	2.9	7.2
**BghiP**	0.9	15.4	19.6	49.0
**IP**	0.8	13.6	21.1	52.8
**BaA**	0.8	13.6	11.5	28.9
**Chry**	1.1	20.0	18.3	45.7
**Σ_{11}PAH**	12.2	215.9	155.2	388.0

Table S3. Model NMAHs concentrations prepared for *in vitro* determination of the inhibition of the transport activity of the zebrafish **Oct1** organic cation transporter. The concentration of NMAH for which a decrease in the passive fluorescence in the zero minute was noticed is marked with *.

NMAHs concentrations (μM)							
**4NP**	**2M4NP**	**3M4NP**	**4NC, 4NG**	**3M4NC, 4M5NC**	**3M5NC**	**2,4DNP**	**4NS**
10	1	25	1	3	3	10	10
25	3	50	10	10	10	50	50
50	10	100	50	25	25	100*	100
100	50*	200*	75*	50	50	150	200
200*	100	300	100	100*(3M4NC)	100*	250	300
400	250	400	200	250*(4M5NC)	200	500	500

NMAHs concentrations (mg/L)								
**4NP**	**2M4NP**	**3M4NP**	**4NC**	**4NG**	**3M4NC, 4M5NC**	**3M5NC**	**2,4DNP**	**4NS**
1.4	0.2	3.8	0.2	0.2	0.5	0.5	1.8	2.0
3.5	0.5	7.7	1.6	1.7	1.7	1.7	9.2	10.0
7.0	1.5	15.3	7.8	8.5	4.2	4.2	18.4	19.9
13.9	7.7	30.6	11.6	12.7	8.5	8.5	27.6	39.8
27.8	15.3	45.9	15.5	16.9	16.9	16.9	46.0	59.8
55.6	38.3	61.3	31.0	33.8	42.3	33.8	92.1	99.6

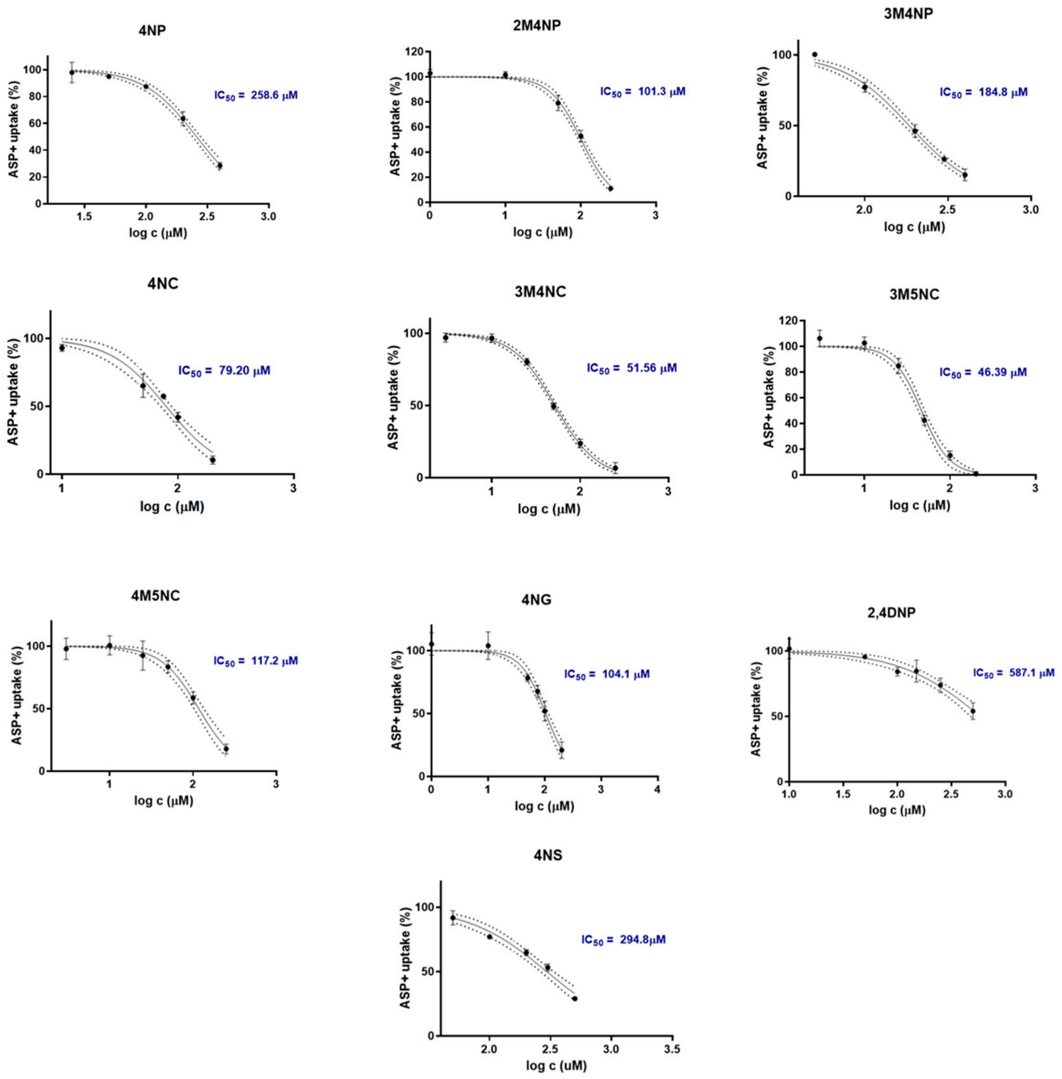

Figure S6. Dose-response curves of the *in vitro* inhibition of the transport activity of the zebrafish organic **cation transporter Oct1** stably expressed in the cell lines Flp-In-293-drOct1 by measuring the inhibition of the uptake of the model substrate ASP+ (%), after incubation with a series of **model NMAHs** concentrations.

Table S4. Model NMAHs concentrations prepared for *in vitro* determination of the inhibition of the transport activity of the zebrafish **Oatp1d1** organic anions transporter.

**NMAHs concentrations (μM)**						
**5NSA, 2,4DNP**	**3NSA**	**4NP, 3M4NC, 4NS 3M5NC, 4M5NC,**	**2M4NP**	**3M4NP**	**4NC**	**4NG**
0.5	0.1	3	1	25	10	10
3	1	10	10	50	25	25
10	10	25	25	100	50	50
25	25	50	50	150	100	100
50	50	100	100	300	300	300
100	100	300	300	500	400	400
300	300	/	/	/	/	/

**NMAHs concentrations (mg/L)**									
**5NSA**	**2,4DNP**	**3NSA**	**4NP**	**3M4NC, 3M5NC, 4M5NC**	**4NS**	**2M4NP**	**3M4NP**	**4NC**	**4NG**
0.1	0.1	0.02	0.4	0.5	0.6	0.2	3.8	1.6	1.7
0.5	0.6	0.18	1.4	1.7	2.0	1.5	7.7	3.9	4.2
1.8	1.8	1.8	3.5	4.2	5.0	3.8	15.3	7.8	8.5
4.6	4.6	4.6	7.0	8.5	10.0	7.7	23.0	15.5	16.9
9.2	9.2	9.2	13.9	16.9	19.9	15.3	45.9	46.5	50.7
18.3	18.4	18.3	41.7	50.7	59.7	45.9	76.6	62.0	67.7
54.9	55.2	54.9	/	/	/	/	/	/	/

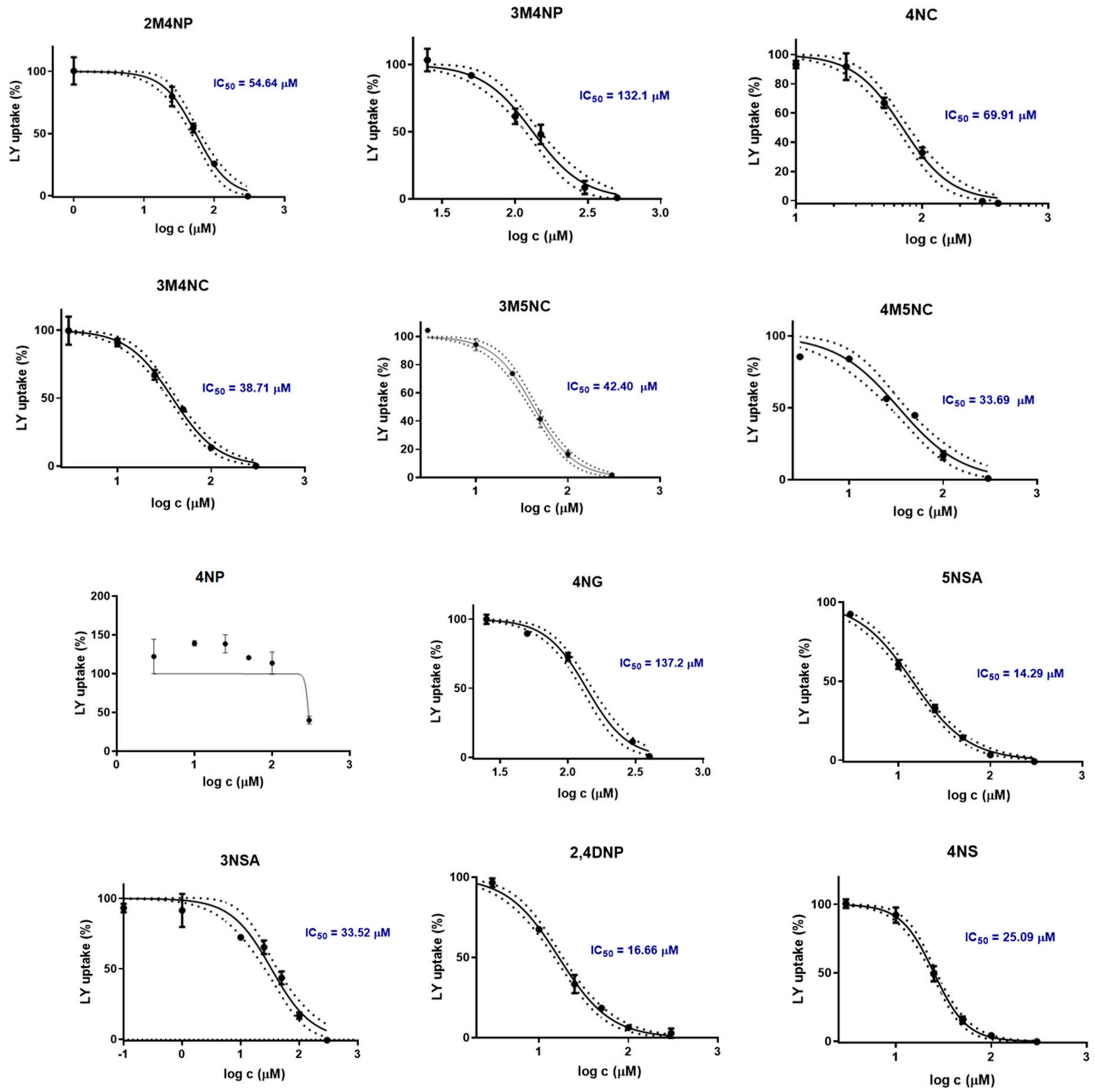

Figure S7. Dose-response curves of the *in vitro* inhibition of the transport activity of the zebrafish organic **anion transporter Oatp1d1** stably expressed in the cell lines Flp-In-293-drOatp1d1 by measuring the inhibition of the uptake of the model substrate and LY (%) after incubation with a series of **model NMAHs** concentrations.

Table S5. Concentrations of **NMAHs mixtures** prepared for *in vitro* determination of the inhibition of the transport activity of the zebrafish **Oatp1d1** organic anions transporter and **Oct1** organic cation transporter.

**Model NMAHs mixtures (mg/L)**	
**10 NMAHs**	**5 NMAHs**
0.5	0.5
1.0	1.0
2.6	2.5
3.4	3.3
10.3	9.9
25.8	24.9

A

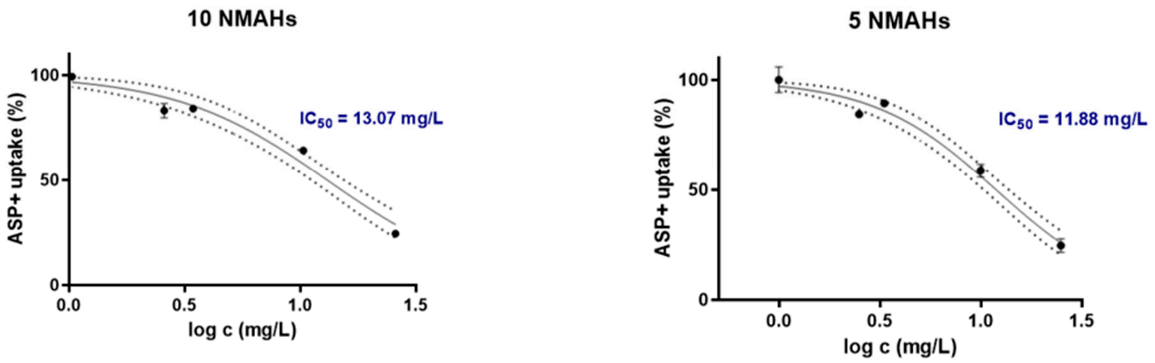

B

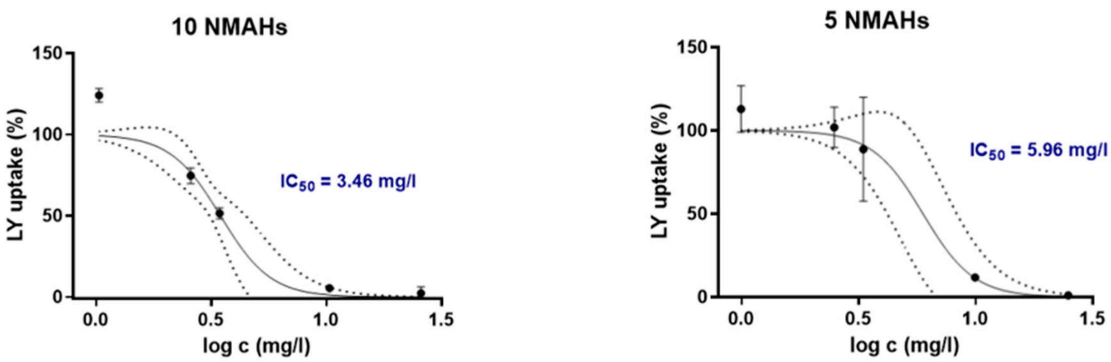

Figure S8. Dose-response curves of the *in vitro* inhibition of the transport activity of the zebrafish organic cation transporter **Oct1 (A)** and organic anion transporter **Oatp1d1 (B)** after incubation with a **NMAHs mixtures** (10 NMAHs and 5 NMAHs) concentrations.

Table S6. Model NMAHs concentrations prepared for *in vitro* determination of induction potential of CYP1A1 detoxification enzymes by measuring the ethoxyresorufin-O-deethylase (**EROD**) activity in PLHC-1 fish cells.

NMAHs concentrations (µM)
**4NP, 2M4NP, 3M4NP, 4NG, 5NSA, 3NSA, 2,4DNP, 3M4NC, 3M5NC, 4M5NC, 4NS, 4NC**
1
3
10
30
100
300

NMAHs concentrations (mg/L)							
**4NP**	**2M4NP, 3M4NP**	**4NG**	**5NSA, 3NSA**	**2,4DNP**	**3M4NC, 3M5NC, 4M5NC**	**4NS**	**4NC**
0.1	0.2	0.2	0.2	0.2	0.2	0.2	0.2
0.4	0.5	0.5	0.6	0.6	0.5	0.6	0.5
1.4	1.5	1.7	1.8	1.8	1.7	2.0	1.6
4.2	4.6	5.1	5.5	5.5	5.1	6.0	4.6
13.9	15.3	16.9	18.3	18.4	16.9	19.9	15.5
41.7	45.9	50.7	54.9	55.2	50.7	59.7	46.5

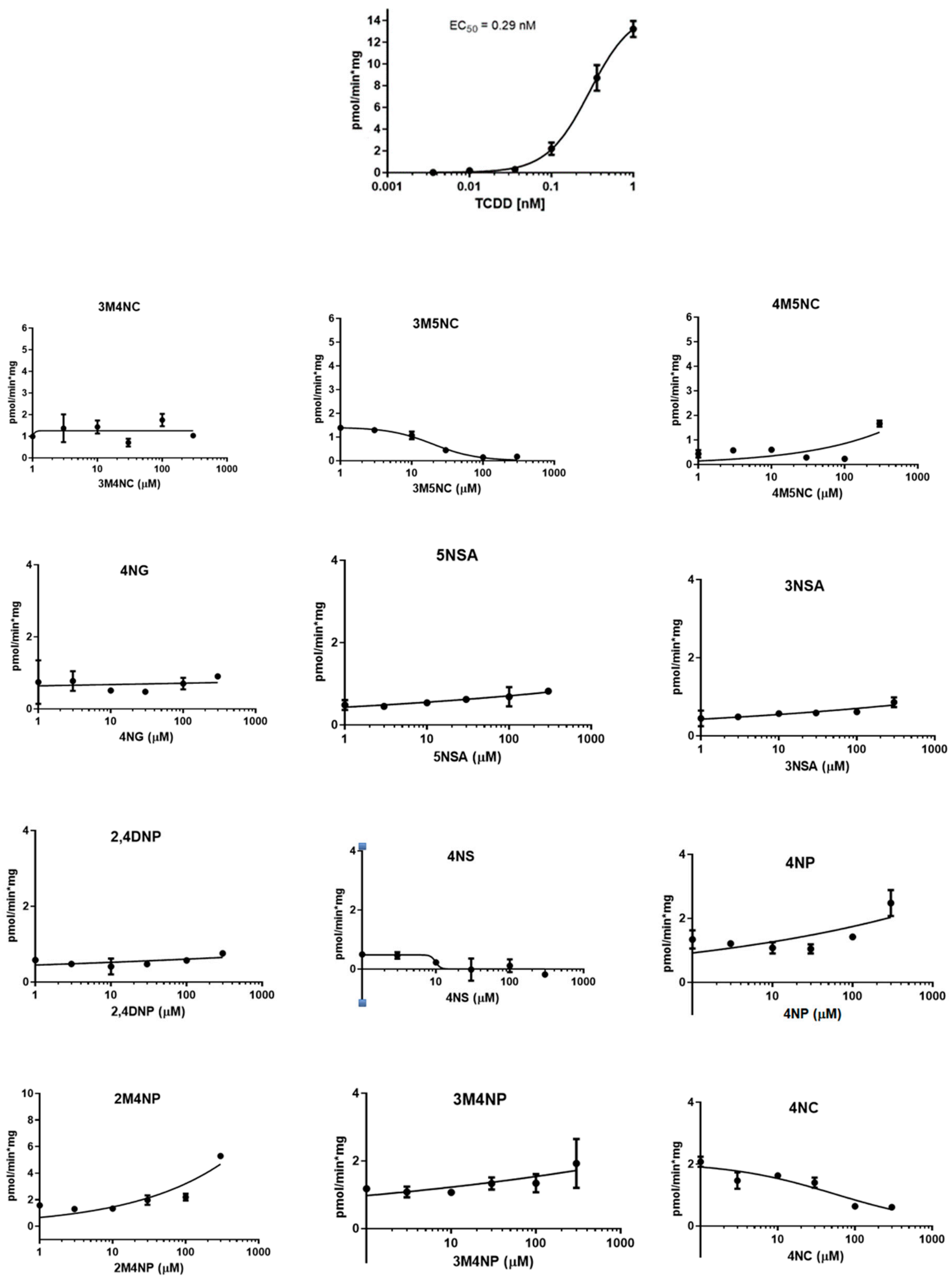

Figure S9. EROD bioassay for model 2,3,7,8-tetrachlorodibenzo-p-dioxin (TCDD) and tested **NMAHs.**

Table S7. Concentrations of **model NMAHs mixtures** for *in vitro* determination of induction potential of CYP1A1 detoxification enzymes by the **EROD** biassay.

**Model NMAHs mixtures (mg/L)**	
**10 NMAHs**	**5 NMAHs**
0.5	0.5
1.0	1.0
2.6	2.6
3.4	3.4
10.3	10.3
25.8	24.9

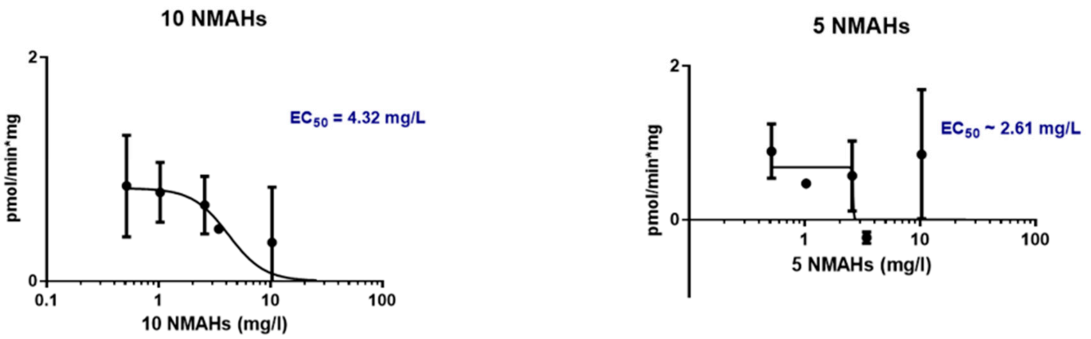

Figure S10. EROD bioassay for the **mixture of 10 NMAHs and 5 NMAHs.**

Table S8. Model NMAHs concentrations prepared for *in vitro* determination of the acute cytotoxic effect on fish PLHC-1 cells by the **MTT assay.**

**NMAHs concentrations (μM)**		
**4NP, 2M4NP, 3M4NP, 4NG, 5NSA, 3NSA, 2,4DNP, 3M4NC, 3M5NC, 4M5NC**	**4NS**	**4NC**
1	0.5	1
3	1	3
10	5	10
30	10	30
100	25	100
300	50	400

**NMAHs concentrations (mg/L)**							
**4NP**	**2M4NP, 3M4NP**	**4NG**	**5NSA, 3NSA**	**2,4DNP**	**3M4NC, 3M5NC, 4M5NC**	**4NS**	**4NC**
0.1	0.2	0.2	0.2	0.2	0.2	0.1	0.2
0.4	0.5	0.5	0.6	0.6	0.5	0.2	0.5
1.4	1.5	1.7	1.8	1.8	1.7	1.0	1.6
4.2	4.6	5.1	5.5	5.5	5.1	2.0	4.6
13.9	15.3	16.9	18.3	18.4	16.9	5.0	15.5
41.7	45.9	50.7	54.9	55.2	50.7	10.0	62.0

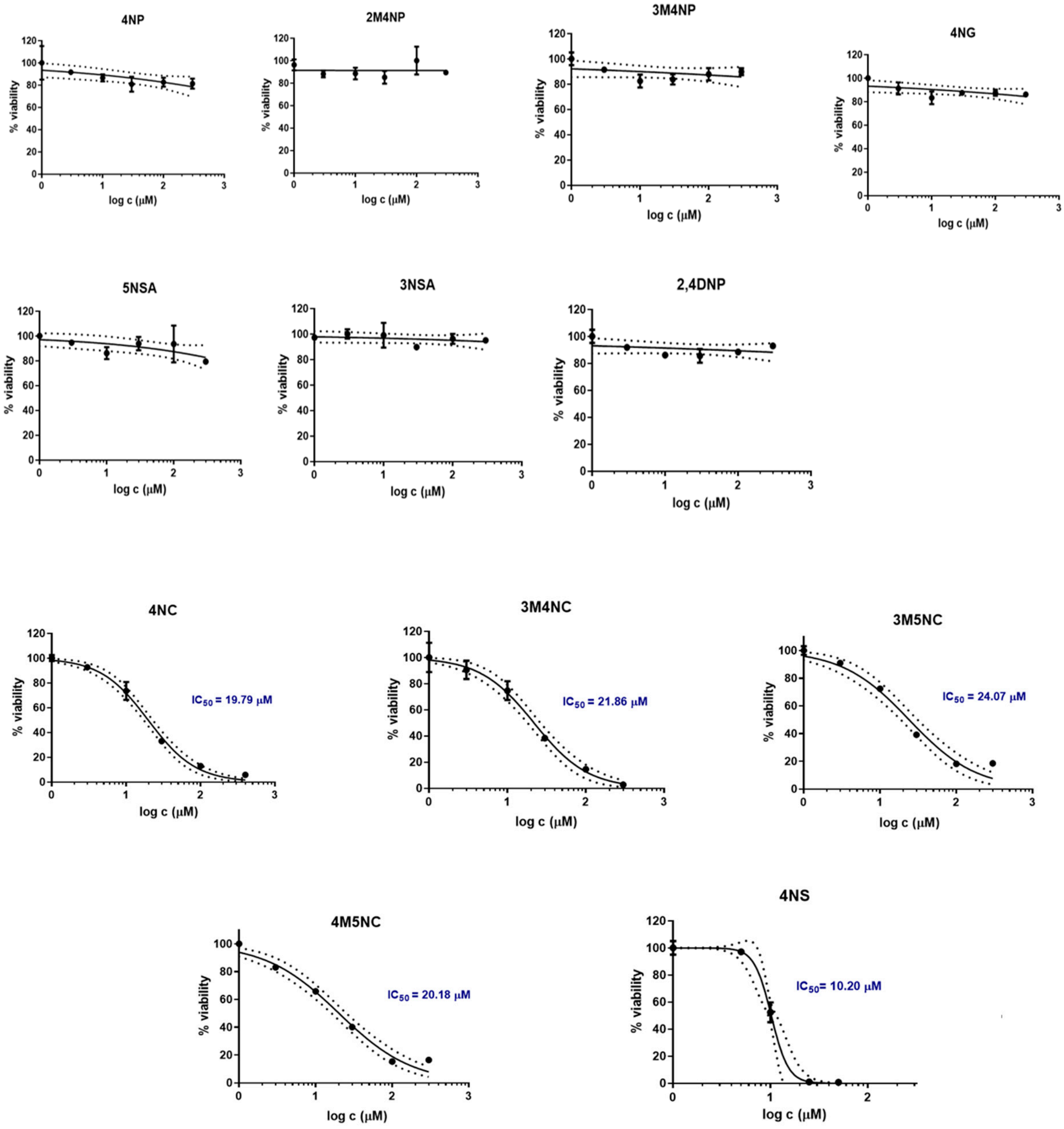

Figure S11. *In vitro* determination of the acute cytotoxic effect of individual **model NMAHs** by the **MTT assay** after exposure to NMAHs concentrations for 72 hours.

Table S9. Model NMAHs concentrations prepared for *in vivo* determination of chronic toxic effects on freshwater green algae *Scenedesmus subspicatus* by the **AlgaeTox assay.**

**NMAHs concentrations (μM)**						
**4NP, 2,4DNP**	**2M4NP, 3M4NP**	**4NC**	**3M4NC, 3M5NC, 4M5NC**	**4NG**	**5NSA, 3NSA**	**4NS**
100	25	1	1	25	100	10
150	50	1.5	1.5	50	150	25
200	100	3	3	75	200	50
300	150	5	4.5	100	250	75
400	200	8	6	150	300	100
500	300	10	9	300	600	300

**NMAHs concentrations (mg/L)**							
**4NP**	**2,4DNP**	**2M4NP, 3M4NP**	**4NC**	**3M4NC, 3M5NC, 4M5NC**	**4NG**	**5NSA, 3NSA**	**4NS**
13.9	18.4	3.8	0.2	0.2	4.2	18.3	2.0
20.9	27.6	7.7	0.3	0.3	8.5	27.5	5.0
27.8	36.8	15.3	0.5	0.5	12.7	36.6	10.0
41.7	55.2	23.0	0.8	0.8	16.9	45.8	14.9
55.6	73.6	30.6	1.2	1.0	25.4	54.9	19.9
69.6	92.1	45.9	1.6	1.5	50.7	109.9	59.8

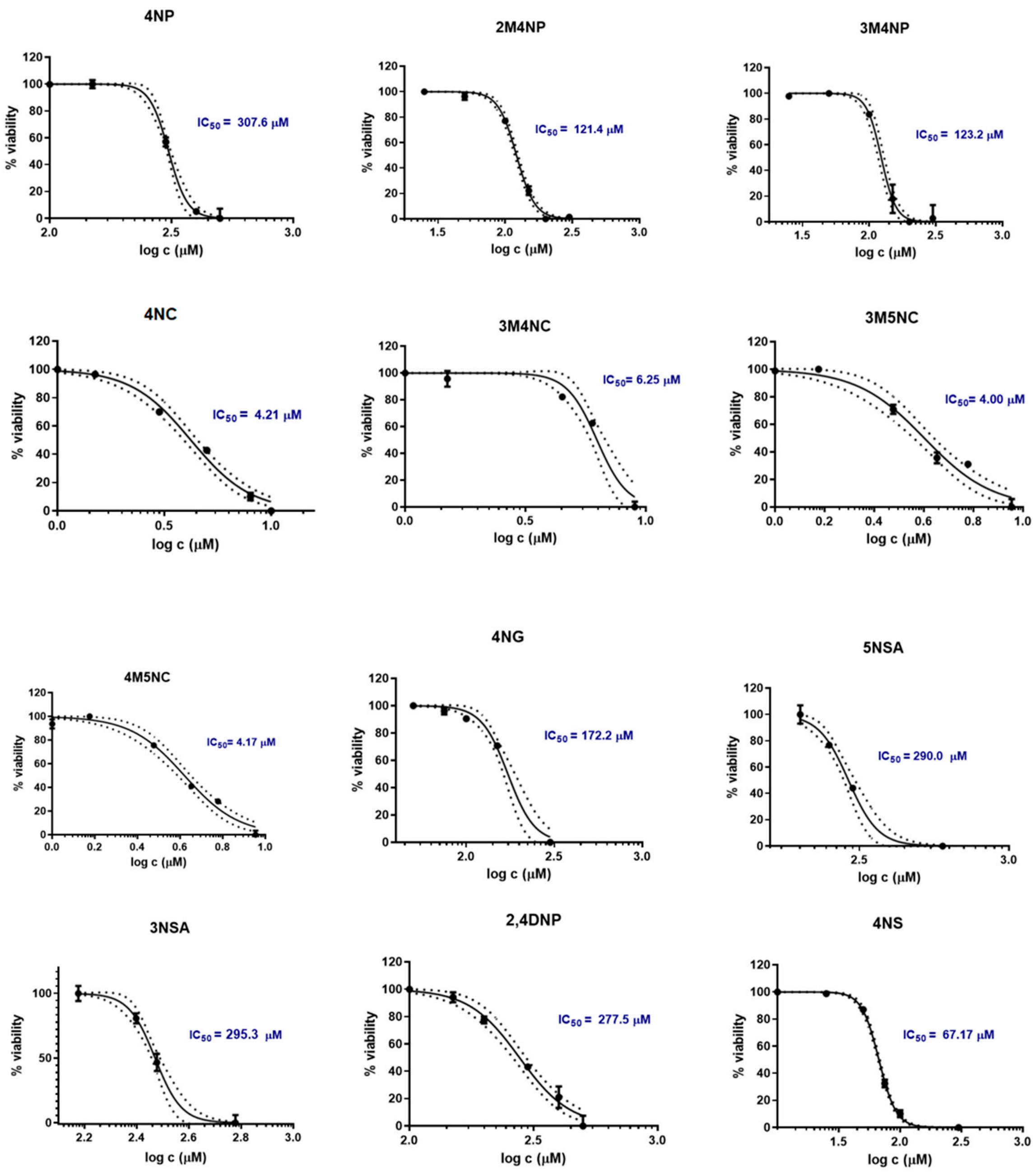

Figure S12. Results of i*n vivo* determination of chronic toxic effects of individual **model NMAHs** by the **AlgaeTox test** after 96-hour exposure to a range of NMAHs concentrations.

Table S10. Concentrations of **model NMAHs mixtures** prepared for in vitro determination of acute and in vivo determination of chronic toxic effects by the **MTT and AlgaeTox test**, respectively.

**Model NMAHs mixtures (mg/L)**			
**MTT test**		**AlgaeTox test**	
10 NMAHs	5 NMAHs	10 NMAHs	5 NMAHs
2.2	2.1	1.0	1.0
3.2	3.1	2.6	2.5
6.4	6.2	3.4	3.3
12.9	12.4	10.3	9.9
25.8	24.9	25.8	24.9

A

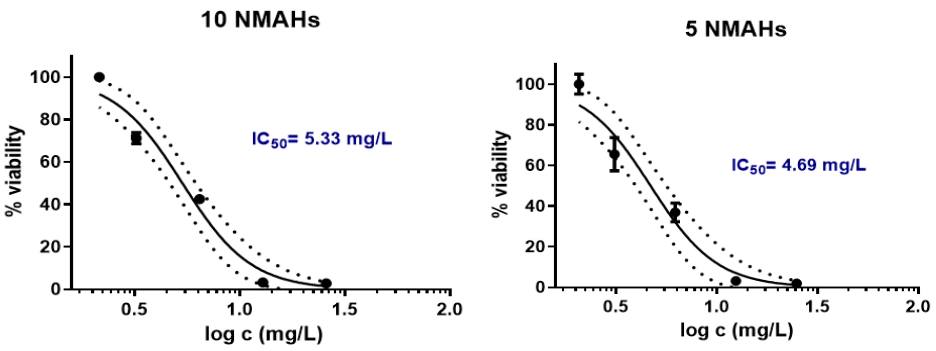

B

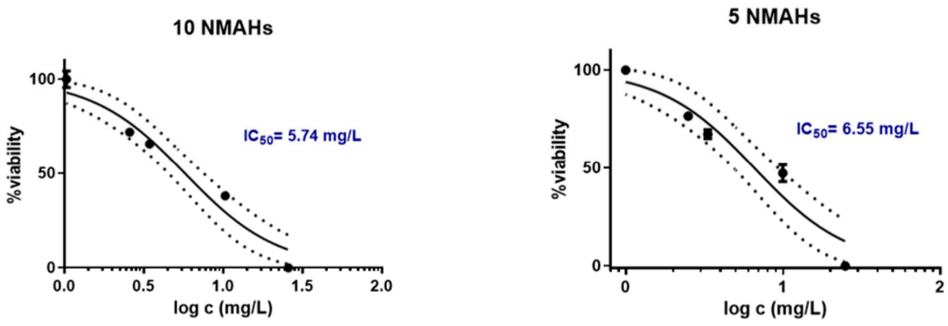

Figure S13. Results of determination of acute (**A**) and chronic (**B**) toxic effects of NMAHs mixtures (**10 NMAHs and 5 NMAHs**) by the **MTT test** and **AlgaeTox test**, respectively.
